# A Multiomics Perspective on Plant Cell Wall-Degrading Enzyme Production: Insights from the Unexploited Fungus *Trichoderma erinaceum*

**DOI:** 10.3390/jof10060407

**Published:** 2024-06-05

**Authors:** Michelle A. de Assis, Jovanderson J. B. da Silva, Lucas M. de Carvalho, Lucas S. Parreiras, João Paulo L. F. Cairo, Marina P. Marone, Thiago A. Gonçalves, Desireé S. Silva, Miriam Dantzger, Fernanda L. de Figueiredo, Marcelo F. Carazzolle, Gonçalo A. G. Pereira, André Damasio

**Affiliations:** 1 Laboratory of Enzymology and Molecular Biology (LEBIMO), Department of Biochemistry and Tissue Biology, Universidade Estadual de Campinas (UNICAMP), Campinas 13083-862, São Paulo, Brazil; mialexandrino@gmail.com (M.A.d.A.); joao.lourencofrancocairo@york.ac.uk (J.P.L.F.C.); tikogoncalvesta@gmail.com (T.A.G.); fernandalfigueiredo@gmail.com (F.L.d.F.); 2 Genomics and BioEnergy Laboratory (LGE), Department of Genetics, Evolution, Microbiology and Immunology, Universidade Estadual de Campinas (UNICAMP), Campinas 13083-862, São Paulo, Brazil; jovanderson14@hotmail.com (J.J.B.d.S.); lucasmigueel@gmail.com (L.M.d.C.); lucparreiras@gmail.com (L.S.P.); mdantzger@gmail.com (M.D.); marcelo.carazzolle@gmail.com (M.F.C.); goncalo@unicamp.br (G.A.G.P.); 3 York Structural Biology Laboratory (YSBL), Department of Chemistry, University of York, York YO10 5DD, UK; 4 SENAI Institute for Biomass Innovation, Três Lagoas 79640-250, Brazil; d.silva@ms.senai.br

**Keywords:** *Trichoderma*, hydrolysis, lignocellulose, multiomics, beta-glucosidase

## Abstract

*Trichoderma erinaceum* is a filamentous fungus that was isolated from decaying sugarcane straw at a Brazilian ethanol biorefinery. This fungus shows potential as a source of plant cell wall-degrading enzymes (PCWDEs). In this study, we conducted a comprehensive multiomics investigation of *T. erinaceum* to gain insights into its enzymatic capabilities and genetic makeup. Firstly, we performed genome sequencing and assembly, which resulted in the identification of 10,942 genes in the *T. erinaceum* genome. We then conducted transcriptomics and secretome analyses to map the gene expression patterns and identify the enzymes produced by *T. erinaceum* in the presence of different substrates such as glucose, microcrystalline cellulose, pretreated sugarcane straw, and pretreated energy cane bagasse. Our analyses revealed that *T. erinaceum* highly expresses genes directly related to lignocellulose degradation when grown on pretreated energy cane and sugarcane substrates. Furthermore, our secretome analysis identified 35 carbohydrate-active enzymes, primarily PCWDEs. To further explore the enzymatic capabilities of *T. erinaceum*, we selected a β-glucosidase from the secretome data for recombinant production in a fungal strain. The recombinant enzyme demonstrated superior performance in degrading cellobiose and laminaribiose compared to a well-known enzyme derived from *Trichoderma reesei*. Overall, this comprehensive study provides valuable insights into both the genetic patterns of *T. erinaceum* and its potential for lignocellulose degradation and enzyme production. The obtained genomic data can serve as an important resource for future genetic engineering efforts aimed at optimizing enzyme production from this fungus.

## 1. Introduction

The circular economy and integrated biorefineries have gained attention in recent decades due to the valorization of the lignocellulosic biomass derived from the plant cell wall (PCW) [1,2,3]. This material represents the most abundant renewable resource on Earth [4], being composed of cellulose fibers embedded within a matrix of hemicelluloses, pectin, and lignin [5]. These polysaccharides can be used as feedstock after their conversion into monomers such as glucose (C6) and xylose (C5) in several bioprocesses [2,6]. The complex and resistant structure found in lignocellulose requires processes such as pretreatment and enzymatic digestion to release its monomers. The presence of lignin and hemicellulose in the structure poses a challenge regarding enzymatic accessibility to the main cellulose fibrils [7,8]. Lignin is a significant inhibitor of hydrolysis due to its nonproductive adsorption of enzymes, highlighting the need for improved and efficient enzymatic cocktails containing plant cell wall-degrading enzymes (PCWDEs) for this process [9].

Among the PCWDEs involved in lignocellulosic biomass breakdown, some of them are classified as cellulases, a group that includes endoglucanases (EGs) (EC 3.2.1.4) and cellobiohydrolases (CBHs), enzymes responsible for cleaving β-1,4-glycosidic linkages in amorphous regions or cleaving reducing ends (EC 3.2.1.176) and nonreducing ends (EC 3.2.1.91) releasing cellobiose [10], respectively. Similarly, for hemicellulose catalysis, the endo-1,4-β-D-xylanases (XLNs) (EC 3.2.1.32) hydrolyze β-1,4 linkages, promoting the formation of xylooligosaccharides (XOs), while β-1,4-D-xylosidases (BXLs) (EC 3.2.1.37) release xylose from XOs’ nonreducing ends. In addition, β-1,4-D-mannanases (MANs) cleave internal linkages, converting mannooligosaccharides to mannose; acetyl xylan esterases (AXEs) cleave carboxylic esters; and α-L-arabinofuranosidases (ABFs) catalyze the hydrolysis of arabinofuranoside linkages. Enzymes such as α-galactosidase (AGL), α-glucuronidase (AGU), and feruloyl esterase (FAE) are also responsible for removing the side chain groups present in the hemicellulose structure [11,12,13,14]. Moreover, lytic polysaccharide monooxygenases (LPMOs) are copper-dependent enzymes that act through an oxidative mechanism to disrupt crystalline cellulose (EC 1.14.99.54 and 1.14.99.56), chitin, xylan, xyloglucan, glucomannan, lichenan, β-glucan, starch, or hemicellulose by starting structural breakage and boosting the activity of the other enzymes via the introduction of new chain ends [15,16,17,18,19]. All these enzymes can be classified according to their amino acid sequence and structural similarity according to the carbohydrate-active enzymes (CAZymes) database. They are grouped into families of glycoside hydrolases (GHs), carbohydrate esterases (CEs), polysaccharide lyases (PLs), glycosyltransferases (GTs), and auxiliary activities (AAs) [20].

Many fungal species can decompose lignocellulosic biomass efficiently via the secretion of PCWDEs, and most of the commercial enzymatic cocktails are composed of fungal-derived enzymes [21,22,23,24,25,26]. The genus *Trichoderma* is a well-known group of ascomycetes that includes a broad spectrum of biotypes ranging from soil colonizers to symbionts in plants. Some species are commonly used as a platform for lignocellulolytic enzyme production [27,28,29]. *Trichoderma reesei*, after decades of genetic modifications and improvements, has become the most robust microbial cell factory for industrial cellulase production, representing *Trichoderma* strains’ biodegradation potential and substrate colonization performance [30,31]. Nowadays, *T. reesei* is described as a high-performance microbial cell factory for lignocellulolytic enzyme production, producing 100 g/L of secreted protein [32,33,34].

The production of cellulases and hemicellulases by *T. reesei* is influenced by the composition of the media. The fungus produces high levels of enzymes in the presence of cellulose, complex plant material, cellobiose, lactose, or sophorose. Conversely, enzyme production is lower in the presence of glucose, fructose, or glycerol [35,36]. The major cellulase and hemicellulase genes reported are regulated according to the availability of carbon sources. Some substrates induce the expression of PCWDEs, while glucose acts as a repressing carbon source. This regulation is mediated by transcription factors (TFs), including the repressors CREI and ACEI; the activators ACEII, ACEIII, and XYRI; and the CCAAT-binding complex Hap 2/3/5 [37,38,39,40,41]. The medium’s composition also influences sugar transport, with many transporter genes being upregulated in the presence of cellulase-inducing carbon sources and regulated by the same set of TFs involved in cellulase production [42]. Additionally, deleting specific transporters affects enzyme production, sugar transport, and *T. reesei* growth. While about 50–100 genes code for sugar transporters, many of which belong to the major facilitator superfamily (MFS), only a limited number of these genes have been characterized [43].

Considering that around 120,000 fungal species have currently been cataloged, the number of fungal strains available for enzyme production at industrial levels is limited, emphasizing the importance of the bioprospection of alternative fungal cell factories [44]. A *Trichoderma erinaceum* strain was isolated from decaying sugarcane straw samples in a second-generation (2G) ethanol plant, and the secretomes produced on a pretreated sugarcane straw (SCS)-containing medium showed higher specific activities of XLNs and β-glucosidases (BGLs) than the *T. reesei* CBS 130855 strain. Moreover, *T. erinaceum* secretomes applied to pretreated SCS degradation yielded a higher glucose concentration and lower cellobiose accumulation than *T. reesei* secretomes produced under the same conditions [45].

Cellobiose and low-molecular-weight oligomers are the substrates for BGLs, releasing primarily glucose as a product [46,47]. BGLs play a crucial role in the complete hydrolysis of plant biomass. This group of enzymes converts cellobiose and glucan-based oligosaccharides into glucose, providing substrates for yeast fermentation and relieving the cellobiose-mediated inhibition of cellulases (EGs and CBHs) [48,49,50]. The predominant enzymes in *T. reesei*’s secretome are CBHI and CBHII, representing up to 85% of the total cellulases. Although the fungus has an efficient cellulolytic arsenal, its BGL activity is considered limited and one of the bottlenecks in *T. reesei*’s enzymatic cocktail [51,52].

In this study, we investigated *T. erinaceum* by analyzing its genomic characteristics as well as its transcriptome and secretome profiles. The fungus was cultivated using various carbon sources, including glucose (Glc), Avicel^®^ (Avi; microcrystalline cellulose, Merck Life Science, Espoo, Finland), pretreated SCS, and pretreated energy cane bagasse (ECB). We discussed the repertoire of PCWDEs, sugar transporters, and transcription factors involved in polysaccharide metabolism. Additionally, considering the higher activity of BGLs in the secretome of *T. erinaceum* compared to that of *T. reesei* and the low accumulation of cellobiose observed, a BGL enzyme found in the *T. erinaceum’s* secretome was selected for further biochemical characterization.

## 2. Materials and Methods

### 2.1. Strains, Media, and Growth Capacity

The *Trichoderma erinaceum F3* was isolated from samples of decomposing SCS collected at GranBio’s Experimental Station (BioVertis, Barra de São Miguel, Brazil) and registered in the National System for the Management of Genetic Heritage and Associated Traditional Knowledge (Sisgen) number A6997EC. Fungal spores were inoculated in potato dextrose agar (PDA) at 30 °C for approximately 5 days. To analyze fungal development, radial growth was evaluated in PDA or plates containing Mandels–Adreotti (MA) medium [(1.4 g/L (NH_4_)_2_SO_4_; 2.0 g/L KH_2_PO_4_; 0.3 g/L MgSO_4_.7H_2_O; 0.3 g/L CaCl_2_.2H_2_O; 0.3 g/L urea; 1 g/L peptone; 5 mg/L FeSO_4_.7H_2_O; 1.6 mg/L MnSO_4_.H_2_O; 1.4 mg/L ZnSO_4_.7H_2_O; 2 mg/L CoCl_2_.2H_2_O] at pH 4.5, supplemented with 10 g/L of glucose, xylose, lactose, sucrose, cellobiose, maltose, beechwood xylan, corn starch, carboxymethylcellulose (CMC), Avi PH-101, or steam explosion-pretreated SCS. *T. erinaceum* was inoculated using 10^5^ spores in the center of each plate (4 replicates per condition).

### 2.2. Genomic DNA Preparation, Sequencing, and Assembly

*T. erinaceum* spores were suspended in sterile dH_2_O to a final concentration of 10^7^ spores per mL. Then, 100 µL of the spore suspension was inoculated into 100 mL of potato dextrose broth (BD Difco, Franklin Lakes, NJ, USA) and incubated at 28 °C and 200 rpm for 48 h. The fungal culture was centrifuged, and the obtained cell pellet was washed twice with sterile dH_2_O. DNA extraction was conducted as previously described [53]. The extracted gDNA was purified with a Quick-DNA Miniprep kit (Zymo Research, Irvine, CA, USA). The 300 bp paired-end purified DNA sequencing was conducted with an Illumina MiSeq system by a third-party service provider (Helixxa, Paulínia, Brazil).

The quality of the generated reads was assessed with FastQC v. 0.11.8 [54]. Reads were trimmed and filtered by size (>150 bp) with Trimmomatic v. 0.36 [55] and assembled with SPAdes v. 3.15.5 [56] using multiple k-mer sizes (25, 45, 65, 85, 105, 125, 127). Gene prediction was performed with GeneMarker v. 4.6 with parameter “--min_contig 1000” [57], and genome integrity was assessed with BUSCO v. 4.1.2 using the hypocreales_odb10 database [58].

### 2.3. Orthogroup Identification and Gene Family Expansion Analysis

To study protein evolution among *Trichoderma* species, we used the proteomes of *T. erinaceum* and other species (*T. asperellum*, *T. atroviride*, *T. citrinoviride*, *T. harzianum*, *T. longibrachiatum*, *T. reesei*, and *T. virens*) available in Ensembl Fungi (http://fungi.ensembl.org/) (accessed on 13 February 2023). *Chaetomium globosum* was set as an outgroup. Orthogroups were identified with OrthoFinder v.2.5.2 [59], and the single-copy orthogroups were used for phylogeny construction. Protein sequences within each orthogroup were aligned with MAFFT v. 7.520 [60] with parameter “--globalpair --maxiterate 1000”. We used AMAS [61] to concatenate all alignments in a supertree, which was used as input to IQTREE v. 2.2.6 [62] with parameter “-b 1000 -m TEST”. The gene count data file generated by OrthoFinder was used for the analysis of gene family evolution with CAFE v. 5.0.0 [63], with a *p*-value = 0.05, and we tested λ values (parameter “-k”) 0.01, 0.02, 0.2, and 0.5. We used λ = 0.02 due to the model adjustment.

### 2.4. Submerged Cultivation with Different Carbon Sources

Spores of the *T. erinaceum* were thawed, streaked onto PDA medium, and incubated at 30 °C until sporulation. From the resulting plates, spores were suspended in 0.1% Triton X-100 to a final concentration of 10^7^ spores per mL. Then, 500 µL of the spore suspension was inoculated into shake flasks containing 200 mL of MA medium. The MA medium was supplemented with 10 g/L of the following carbon sources: steam-explosion-pretreated SCS and ECB, Avi, or Glc (composition described in Table S1).

All cultures were incubated in a rotary shaker at 30 °C and 200 rpm. Glc and Avi cultures were cultivated for 72 h, while SCS and ECB cultures were incubated for 144 h. The cultures were then filtered, and fungal biomass was scraped into a 50 mL falcon tube and flash-frozen with liquid nitrogen. Experiments were conducted in three biological replicates.

### 2.5. Transcriptomics Analysis

Frozen hyphae were ground in a ball mill. Liquid nitrogen was constantly added to the samples to avoid melting throughout the grinding procedure. RNA was extracted from the powdered hyphae with an RNeasy Plant Mini Kit (Qiagen, Hilden, Germany). Quality analysis of the purified RNA and 150 bp paired-end sequencing with an Illumina HiSeq 2500 was conducted by a third-party service provider (Helixxa, Paulínia, Brazil).

The overall quality of the generated reads was assessed with FastQC v. 0.11.8, while contamination and the proportion of ribosomal RNA in the samples were determined with SortMeRNA v. 4.3.6 [64]. Reads from each sample were mapped against the previously assembled reference genome using HISAT2 set to default parameters v. 2.2.1 [65]. The transcriptome was then assembled using StringTie v. 2.2.1, and set to default parameters [66]. The generated gtf files were processed with gffread v. 0.12.7 for extracting nucleotide sequences [66]. Open reading frames (ORFs) were predicted with TransDecoder v. 5.5.0. Gene expression in transcripts per million (TPM) was calculated using Kallisto v. 0.48.0 [67]. Only genes presenting an average expression higher than 5 TPM in at least one of the evaluated conditions were considered for further analysis. Genes with similar expression profiles between treatments were grouped with Clust v. 1.10.8 [68]. Gene set enrichment analysis within gene clusters was performed with topGO v. 4.2 [69].

The identification of genes encoding CAZymes was performed with dbCAN v. 2.0.11 [70]. The list of identified CAZymes was submitted to SignalP v. 5.0 [71] to identify secreted enzymes. Lastly, genes encoding transcription factors were identified with annotation from the CDD database. The identified genes were clustered based on their expression profiles using Clust. Expression data for genes predicted to encode CAZymes targeted to the secretory pathway were log-transformed, normalized, and hierarchically clustered by correlation with single linkage using Gene Cluster 3.0 [72]. The *T. erinaceum F3* genome and transcriptome are available in NCBI.

### 2.6. Fungal Secretomes Preparation

The secretome samples were collected from the same cultivation described in Section 2.4. Considering the faster growth on Glc and Avi, supernatants were collected at 72 h, while, in media containing plant biomass, supernatants were collected at 96, 120, or 144 h after inoculation [73]. The secretomes were isolated by filtration in Miracloth^®^ (Merck Millipore, Darmstadt, Germany) and applied for enzymatic assays or concentrated using Vivaspin^®^ 15 columns (Sartorius, Gottingen, Germany) with a molecular weight cut-off (MWCO) of 10 kDa for proteomic experiments.

### 2.7. Mass Spectrometry

The quantification of proteins from *T. erinaceum* secretomes was performed using the Bradford method [74], with bovine serum albumin solution (BSA) as the reference protein. For further analysis, 10 µg of protein of each sample described in Section 2.6 (24 samples) was alkylated, reduced, digested using trypsin solution, and desalted [75]. A 5 μL aliquot of each sample was analyzed on an Orbitrap Velos ETD mass spectrometer (Thermo Fisher Scientific, Waltham, MA, USA) provided by the Brazilian Biosciences National Laboratory (Brazilian Centre for Research in Energy and Materials). The peptides were separated using a PicoFrit analytical column (20 cm × 75 μm, particle size 5 μm, New Objective) in a flow of 200 nL/min for 85 min. All instrument methods were configured in data-dependent acquisition mode in full-scan MS spectra (m/z 300–1600). The resolution on the Orbitrap was adjusted to r = 60,000, and the 20 most intense peptide ions with charge ≥ 2 were sequentially isolated and fragmented into the linear trap ion using low-energy ICD.

The spectrum was acquired using Thermo Xcalibur v. 2.1 software (Thermo Fisher Scientific, Waltham, MA, USA), and the files with preliminary data were converted to a peak list with Mascot Distiller v. 2.3.2.0 software (Matrix Science, London, UK) using MASCOT v. 2.3.01 (Matrix Science, London, UK). The parameters were lost cleavage by trypsin, fixed modification of carbamidomethylation, methionine oxidation as the variable modification, mass tolerance of 1 Da, and tolerance for precursor ions of 10 ppm. Enzyme identifications were performed using a database containing the transcriptome data in the same conditions and processed by Scaffold software Q+/Q+S, with a false discovery rate (FDR) of less than 1% and 5%, respectively, a minimum similarity of 95% for peptides and 99% for proteins, a minimum of 1 unique peptide for the identification of a protein, in addition to the exclusion of identifications found in only 1 of the 3 biological replicates. The sequences were named according to the entry number defined in the transcriptome data.

### 2.8. Enzymatic Activity Assays of T. erinaceum Secretomes

The enzymatic activities were measured by adding 10 μL of secretomes in 40 μL of 50 mM sodium acetate buffer pH 5.5 and 50 μL of 0.5% substrate (m/v) (CMC, β-glucan, beechwood xylan, or wheat arabinoxylan), followed by incubation for 30 min at 50 °C, according to Ghose [76]. Assays with synthetic substrates were performed by adding 10 μL of secretomes in 40 μL of 50 mM sodium acetate buffer at pH 5.5 and 50 μL of 5 mM 4-nitrophenyl β-D-glucopyranoside (*p*NPG) or 4-nitrophenyl β-D-xylopyranoside (*p*NPX). The assays were incubated at 50 °C for 30 min, and the released 4-nitrophenolate (*p*NP) was measured at 405 nm after adding 100 μL of 1 M Na_2_CO_3_. Enzyme activities were converted to µmol of product per minute (U)/mL.

### 2.9. Recombinant Production of T. erinaceum β-Glucosidase TeBgl3C

The enzyme *Te*Bgl3C coding sequence and the most similar sequence from *T. reesei* (*Tr*Cel3B) were cloned via USER cloning with their native signal peptides into a SwaI-digested vector. The pU2211-1 vector harbors an integrative homology site IS1 to *Aspergillus nidulans*, also containing a glyceraldehyde-3-phosphate dehydrogenase (*PgpdA*) promoter, a tryptophan synthase transcription terminator (*TrpC*) [77], and an orotidine-5′-decarboxylase gene (*pyrG*) cassette that was used as a transformation marker. *A. nidulans* A773 (*pyrG89*; *wA3*; *pyroA4*) was obtained from the Fungal Genetic Stock Center (FGSC) and used for cassette genome integration and recombinant expression [78]. Positive isolates were confirmed by colony PCR.

Spore solutions (10^7^ total) from *A. nidulans* recombinant strains were inoculated in 200 mL of minimum medium (MM) (50 mL/L 20× Clutterbuck salts, 1 mL/L 1000× trace elements, 5 g/L tryptone, 10 g/L glucose; 20× Clutterbuck salts: 120 g/L NaNO_3_, 10.4 g/L KCl, 10.4 g/L MgSO_4_.7H_2_O and 30.4 g/L KH_2_PO_4_; 1000× Trace elements: 22 g/L ZnSO_4_.7H_2_O, 11 g/L H_3_BO_3_, 5 g/L MnCl_2_.4H_2_O, 5 g/L FeSO_4_.7H_2_O, 1.6 g/L CoCl_2_.5H_2_O, 1.6 g/L CuSO_4_.5H_2_O, 1.1 g/L Na_2_MoO_4_.4H_2_O, and 50 g/L Na_2_EDTA), pH 6.5. The cultures were maintained for 36 h at 37 °C for recombinant BGL production.

Fungal mycelium was separated by filtration in Miracloth^®^ (Merck Millipore, Darmstadt, Germany), and the supernatant was loaded into a DEAE Sepharose Fast Flow (GE Healthcare, Darmstadt, Germany) column (20 mL) for ion-exchange chromatography (IEC). The column was previously equilibrated with 100 mM Tris HCl buffer pH 7.5, and the flow rate was set at 1 mL/min. Adsorbed proteins were eluted with NaCl (100 mM to 1 M) in the same buffer. Collected fractions (4 samples with 2 mL each) with detectable enzymatic activity in *p*NPG (fractions collected with 250 mM and 500 mM of NaCl after the IEC) were concentrated to 2 mL using an Amicon Ultra-centrifugal filter 10 MWCO (Millipore, Washington, DC, USA) and further loaded on Superdex200^®^ 16/600 HiLoad (GE Healthcare, Darmstadt, Germany) (124 mL) for size exclusion chromatography (SEC). This second chromatography step was performed using an AKTA^®^ system with a UV detector (280 nm) at a 1 mL/mL flow rate in 50 mM Tris HCl buffer, pH 7.5. The BGLs were eluted with 120 mL of buffer, and 2 mL was collected for each fraction. Three fractions with detectable enzymatic activity in *p*NPG (6 mL) were filtered to 1 mL using an Amicon Ultra-centrifugal filter 10 MWCO (Millipore, Washington, DC, USA). Protein profiles were analyzed via SDS-PAGE, and the concentration was determined with a BCA protein assay kit (Thermo Scientific, Rockford, IL, USA). Biochemical characterization was performed using the purified proteins.

### 2.10. Characterization of TeBgl3C

A three-dimensional model of *Te*Bgl3C (without the signal peptide) was obtained using the I-TASSER server without a reference structure [79]. The C scores, TM scores, and root mean square deviation (RMSD) were calculated for the modeled structure. The model with the highest C scores was used by the I-TASSER server to predict the ligand-binding sites using the COFACTOR [80] and COACH [81] tools.

The biochemical analyses, including substrate specificity, assay temperature and pH, and glucose inhibition, were carried out using purified BGLs (75 ng per reaction). To analyze substrate specificity, we tested several synthetic substrates (4-nitrophenyl derivatives), carboxymethylcellulose (CMC), β-glucan, laminarin, curdlan, cellobiose, laminaribiose, and maltose. Enzymatic activities were measured by adding 10 μL of purified enzyme to 40 μL of 50 mM sodium acetate buffer pH 5.5 and 50 μL of 5 mM of the following substrates: 4-nitrophenyl β-D-glucopyranoside (*p*NPG), 4-nitrophenyl β-D-xylopyranoside (*p*NPX), 4-nitrophenyl α-D-glucopyranoside (*p*NPαG), 4-nitrophenyl α-D-galactopyranoside (*p*NPαGal), 4-nitrophenyl β-D-galactopyranoside (*p*NPGal), or 4-nitrophenyl α-L-arabinofuranoside (*p*NPαAra). The assays were incubated at 50 °C for 30 min, and the released *p*NP was measured at 405 nm after adding 100 μL of 1 M Na_2_CO_3_. For the polysaccharides, CMC, β-glucan, laminarin, and curdlan, the tests were performed by adding 10 μL of purified enzyme to 40 μL of 50 mM sodium acetate buffer pH 5.5 and 50 μL of 0.5% (*m*/*v*) substrate, followed by incubation for 30 min at 50 °C. Reactions with polymeric substrates were stopped with 100 μL of 3,5-dinitrosalicylic acid (DNS) and boiled, and the released reducing sugars were measured at 540 nm. For cellobiose, laminaribiose, and maltose, tests were carried out by adding 10 μL of enzymes in 40 μL of 50 mM sodium acetate buffer at pH 5.5 and 50 μL of 5 mM substrate. After 30 min of incubation at 50 °C, glucose released was measured at 505 nm by adding glucose oxidase and with a peroxidase kit (GOD-PAP method) (Laborlab, Guarulhos, Brazil).

Temperature and pH were evaluated using 10 μL of purified enzyme in 40 μL of 50 mM sodium acetate buffer pH 4.5 and 50 μL of 5 mM *p*NPG. The temperature of incubation varied from 10 to 90 °C. To determine the optimum pH, 0.1 M glycine/sodium phosphate/citric acid buffer (pH 2 to 10) was used, and activity was measured at the optimum temperature previously determined.

A glucose tolerance assay was performed with the same enzyme volume, buffer, and substrate at the previously determined optimum temperature and pH (pH 4.5, 60 °C). Before incubating, 5 to 800 mM of glucose was also added. Enzyme activities were converted to µmol of product generated per minute (U)/protein mg.

### 2.11. Statistical Analysis

Statistical analysis was conducted using *t*-test or ANOVA to compare means among the samples. A significance level of a *p*-value < 0.05 was used for both tests. Results indicated statistically significant differences between groups’ means, supporting the hypotheses tested.

## 3. Results

### 3.1. T. erinaceum Genome Sequencing, Comparative Genomics, and Gene Family Expansion Analysis

The genome of *T. erinaceum* was sequenced with 256-fold coverage using the Illumina MiSeq platform. A total of 222 scaffolds (>1000 bp) were generated by SPAdes. The final genome obtained had a size of 36.17 Mb and N50 of 333,461 bp (34 contigs). Using an ab initio prediction with GeneMarker, 10,942 genes were predicted for the assembled genome. The average gene length was 1481 bp, with an average of 2.73 exons per gene. Genome completeness was assessed by searching for 4494 core genes conserved across the order Hypocreales with BUSCO. The performed analysis predicted a completeness of 99.2% for *T. erinaceum* genome (Figure S1). Given our interest in understanding the potential of *T. erinaceum* to produce PCWDEs, we performed an analysis to specifically identify CAZyme-encoding genes in the assembled genome. This study resulted in 419 genes predicted to encode CAZymes, of which 231 presented a signal peptide directed to the secretory pathway. Of these, 176 were GHs, 20 AAs, 14 CEs, 9 GTs, 7 PLs, and 5 CBMs.

**Figure 1:**
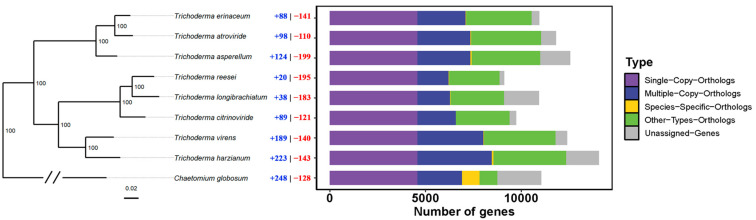
The phylogenetic tree of *T. erinaceum* constructed based on the analysis of single-copy orthologs using OrthoFinder for fungi of the genus *Trichoderma*. The tree was designed using the ggtree package in R. All branches have a bootstrap of 100. The number of expanded families per species is shown in blue, and the contracted families are in red. The horizontal bar graph (right) shows the number of genes for each species and the orthologous classes for each group of genes.

In addition, we did a comparative genomic analysis to identify the shared characteristics and differences of *T. erinaceum* compared to seven other *Trichoderma*. This study of orthologs resulted in 4573 single-copy groups and 9689 orphan genes. Among the orphan genes, 393 were specific to *T. erinaceum*, and 4 orthogroups were exclusive to *T. erinaceum*. Phylogenetic analysis revealed that *T. erinaceum* clustered with *T. atroviride*, and both species were grouped with *T. asperellum*. We identified gene families that underwent expansion or contraction in the genome of *T. erinaceum*, with 88 expanded and 141 contracted families (Figure 1). Among the expanded families, 35 were exclusive to *T. erinaceum*, with 243 genes included. These genes are involved in processes such as amino acid biosynthesis, the shikimate pathway (3-dehydroshikimate dehydratases), hydrolases (ureidoglycolate hydrolase), fungal transcription factors, and other processes related to fungal metabolism (dehydrogenases, DNA binding, methyltransferases, glycolysis, transport protein). We searched for gene families containing CAZymes and transcription factors (TFs) potentially involved in PCWDE regulation in *T. erinaceum*. We found 67 orthogroups with CAZymes and 7 with the TFs (Table S1). Our analysis revealed no discernible copy number variation in *T. erinaceum* for the analyzed genes. This observation suggests that the remarkable versatility shown by this fungus is not only due to variations in gene copy numbers. Instead, our findings evidenced the presence of other regulatory mechanisms that might contribute significantly to its adaptability and functional diversity.

### 3.2. T. erinaceum Nutritional Preferences, Transcriptomics, and Secretome Analysis

**Figure 2:**
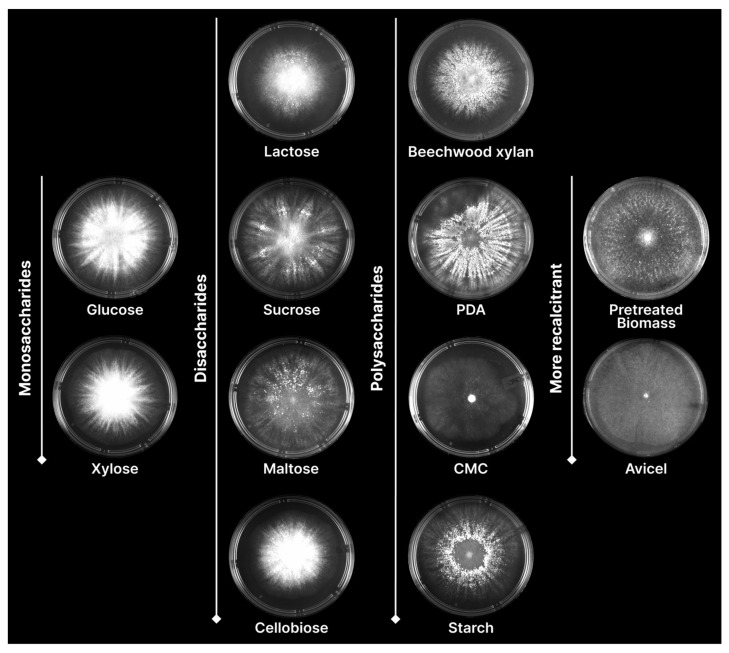
*Trichoderma erinaceum* radial growth on agar plates containing Mandels–Andreotti medium supplemented with different carbon sources after 72 h at 30 °C. Monosaccharides: glucose and xylose; disaccharides: lactose, sucrose, maltose and cellobiose; polysaccharides: beechwood xylan, PDA (potato dextrose agar), CMC (carboxymethylcellulose) and corn starch; more recalcitrant substrates: steam-explosion-pretreated sugarcane straw and Avicel (microcrystalline cellulose PH-101).

The versatility of *T. erinaceum* in utilizing various carbohydrates was observed during its cultivation in MA medium supplemented with monosaccharides, disaccharides, or polysaccharides. *T. erinaceum* growth was detected on all carbon sources analyzed including the monosaccharides glucose and xylose; the disaccharides lactose, sucrose, maltose, and cellobiose; the polysaccharides beechwood xylan, PDA, CMC, and starch; and on recalcitrant substrates such as pretreated SCS and Avi, which demonstrate the capacity to grow on cellulosic and hemicellulosic materials (Figure 2).

To explore the profile of the PCWDEs secreted by *T. erinaceum*, the fungal mycelium and secretomes produced on four different carbon sources were sampled for further analyses. The fungus was cultivated in MA medium supplemented with Glc, Avi, SCS, or ECB (Table S1). The proteome and transcriptome data obtained showed a positive linear correlation coefficient (r) (64% and 56% for SCS and ECB, respectively) based on fold-change (FC) values of the transcripts and proteins identified (glucose × plant biomass condition) (Figure S2).

Transcriptome assembly from *T. erinaceum* samples collected in Glc, Avi, SCS, and ECB generated 24,797 transcripts with open reading frames (ORFs). According to the principal component analysis (Figure S3), expression profiles were divided into two main groups at opposing ends of the PC1 axis, accounting for 75% of the overall variance among the different conditions. These results showed a well-defined separation between samples collected in less complex carbon sources, such as Glc and Avi, from more complex substrates (SCS and ECB). The SCS and ECB profiles are also grouped along the PC2 axis, indicating minor differences in expression patterns between the two conditions. The Glc and Avi expression profiles, although reasonably close along the PC1 axis, are placed on opposite ends of the PC2 axis, demonstrating more significant differences than observed between SCS and ECB.

**Figure 3:**
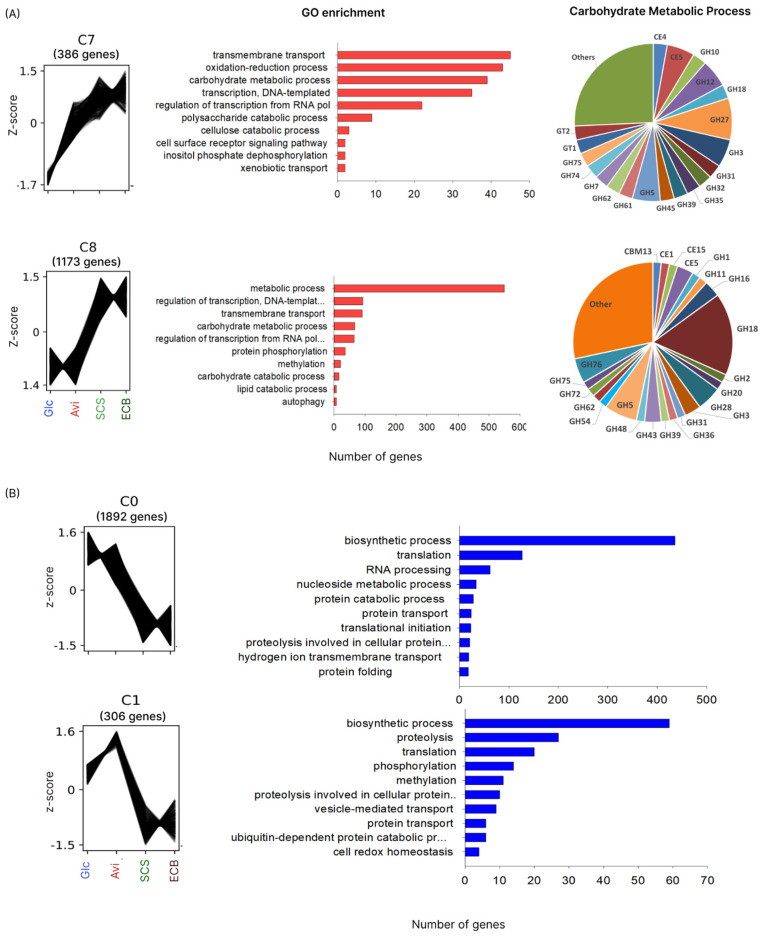
Gene Cluster and Gene Ontology (GO) enrichment analysis. (**A**) GO enrichment of clusters C7 and C8 grouping genes related to biomass degradation focusing on carbohydrate metabolism process and CAZymes families. (**B**) GO enrichment of clusters C0 and C1 with genes overexpressed in Glc and Avi classified mainly into biosynthetic processes. The GO enrichment analysis was performed using the ShinyGO web platform, with a cut-off of FDR ≤ 0.05 for significant biological processes (Glc, glucose; Avi, avicel; SCS, pretreated sugarcane straw; ECB, pretreated energy cane bagasse; GH, glycoside hydrolase; GT, glycosyltransferase; CBM, carbohydrate binding module).

A gene network coexpression analysis was performed using *T. erinaceum* transcriptome data to explore the diversity of CAZymes and PCWDEs. Using the Clust package, we detected 12 coexpression clusters (Figure S4). Clusters C7 and C8 displayed genes enriched in carbohydrate metabolism and contained the largest number of genes encoding plant-biomass-degrading enzymes induced on Avi, SCS, and ECB (C7) or only on SCS and ECB (C8) (Figure 3A). In the C7 cluster (386 genes), genes overexpressed on Avi, SCS, and ECB were enriched for transmembrane transport, oxidation–reduction process, and carbohydrate metabolic process, with 35 genes predicted as CAZymes. C8 was the second largest cluster, with 1173 genes overexpressed in the presence of complex biomass (SCS and ECB) enriched in metabolic process, transcription regulation, transmembrane transport, and carbohydrate metabolic process, with 61 genes encoding CAZymes. Genes exclusively overexpressed in the presence of ECB or SCS were grouped in small clusters, corroborating the difference in the composition of the plant biomass substrates submitted to the same pretreatment. Moreover, genes involved with biosynthetic processes, RNA processing, translation, and protein metabolism were enriched in the presence of Glc and Avi (clusters C0 and C1) (Figure 3B).

**Figure 4:**
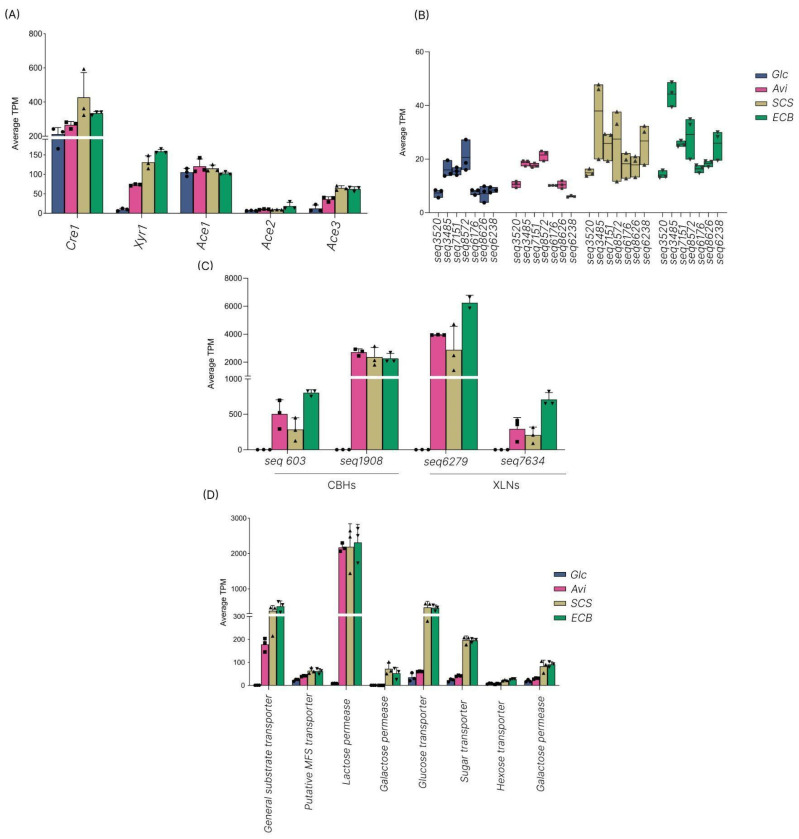
Transcription levels of sugar transporters, transcription factors, and significant CAZymes in an average of transcript per million (TPM). (**A**) Expression levels of five master regulators of PCWDE production: *Cre1*, *Xyr1*, *Ace1*, *Ace2*, and *Ace3*. (**B**) Expression levels of predicted transcription factors were identified in cluster C8 based on the conserved fungal domain (Pfam 04082) with a TPM > 10. (**C**) Expression levels of 4 main enzymes (2 CBHs and 2 XLNs) found in the secretome analysis. (**D**) Expression levels of primary sugar transporters found in cluster C8 based on the pfam 00083 domain (Glc: glucose; Avi: avicel; SCS: pretreated sugarcane straw; ECB: pretreated energy cane bagasse; CBH: cellobiohydrolase; XLN: endo-β-1,4-xylanases). Biological replicates are displayed using the following symbols: circle (Glc), square (Avi), triangle (SCS), and inverted triangle (ECB).

The major TFs potentially involved in PCWDE regulation and sugar transporters were analyzed. The orthologues of genes encoding previously described TFs in *T. reesei* were not statistically classified into a cluster, except for *xyr1* and *cre1*, which were highly expressed in SCS and ECB and clustered in C7 and C8, respectively. *Cre1* showed higher expression in SCS and ECB, as well as *xyr1* and *ace3* (Figure 4A). Additionally, seven predicted TFs were identified in the transcriptome data based on a conserved domain (pfam 04082). The expression profile of these genes was carbon-source-dependent, displaying more transcripts in the presence of SCS and ECB (cluster C8) (Figure 4B). The transcripts of the four main enzymes found in *T. erinaceum* secretomes (seq 603, 1908, 6279, and 7634) were also compared. The predicted CBHs showed higher expression in Avi, ECB, and SCS. Additionally, the expression of gene 6279 (predicted XLN) was similar among SCS, ECB, and Avi (Figure 4C). The predicted sugar transporters were analyzed in clusters C7 and C8 using the conserved domain pfam 00083. A large group of genes classified as “transmembrane transport” was found in cluster C7, within genes encoding sugar transporters induced by Avi, SCS, and ECB. Furthermore, cluster C8 grouped 80 genes related to the same biological process, with 43 annotated as sugar transporters and overexpressed in SCS and ECB. The main putative transporters were annotated as maltose permease, hexose transporter, lactose permease, general substrate transporter, putative MFS transporter, galactose permease, and glucose transporter (Figure 4D).

**Figure 5:**
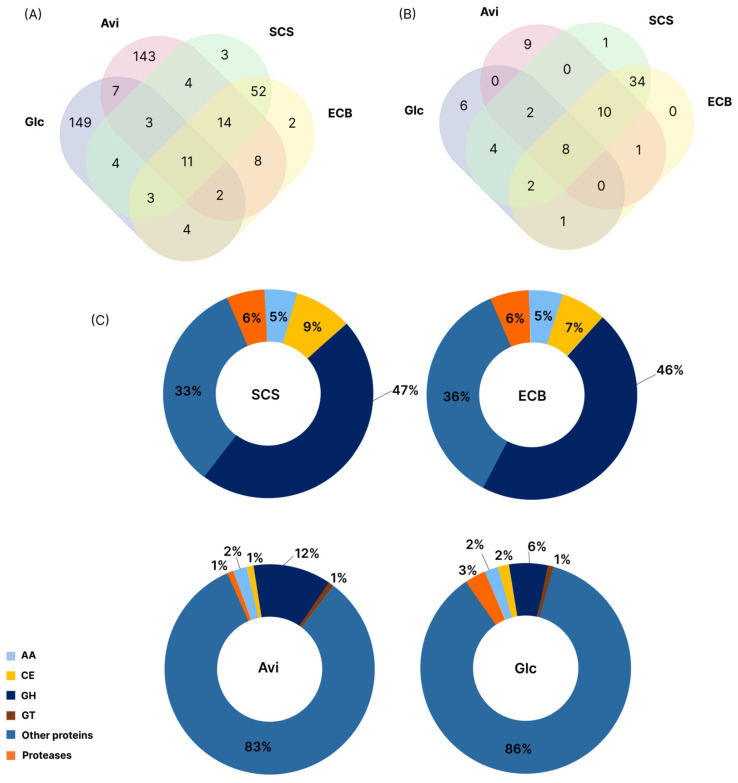
Distribution of proteins and CAZymes identified by mass spectrometry. (**A**) The Venn diagram represents the total number of proteins identified under different conditions (Glucose-Glc, Avicel-Avi, pretreated sugarcane straw-SCS, pretreated energy cane bagasse- ECB). (**B**) Venn diagram representing the number of identified CAZymes. (**C**) Distribution of CAZymes classes (%): Auxiliary Activities (AAs), Carbohydrate Esterases (CEs), Glycoside Hydrolases (GHs), Glycosyltransferases (GTs), “Others” grouping all non-CAZyme proteins and proteases. Steam explosion was used for the pretreatment of SCS and ECB.

To complement the genome and transcriptome data, a secretome analysis was performed to investigate the repertoire of PCWDEs secreted by *T. erinaceum* in the presence of the same substrates. Overall, the number of proteins identified in the secretomes was carbon-source-dependent, with 185 in Glc, 194 in Avi, 97 in ECB, and 95 in SCS. The secretomes produced on Glc and Avi showed a particular pattern compared to the other conditions, with 149 and 143 exclusive proteins, respectively. A total of 11 proteins were identified in all conditions, of which 73% (8) were CAZymes, and 52 (34 CAZymes) were found in SCS and ECB. In addition, 14 proteins were identified in Avi (10 CAZymes) (Figure 5A,B). Although a higher number of proteins were identified on Glc and Avi, SCS and ECB induced a more diverse set of CAZymes (Figure 5C), represented by 47% and 46% of GHs, 9% and 7% of CEs and 5% of AAs, respectively. However, the secretome produced on Avi showed only 12% GHs and 83% proteins classified as “Other”, grouping intracellular proteins not directly related to plant cell wall degradation or high-molecular-weight carbohydrates metabolism. The higher number of PCWDEs found in the secretomes produced on SCS and ECB revealed a conserved mechanism of enzyme induction, including CEs and AAs, for the breakdown of complex substrates.

The production level of each protein was estimated according to the normalized total spectrum count [82]. Two CBHs and two XLNs were differentially abundant in the *T. erinaceum* secretomes produced on SCS or ECB (Figure S5A), representing around 35% and 38% of the total CAZymes. Moreover, even at a lower concentration, PCWDEs important for biomass deconstruction were found in the secretomes produced on SCS, ECB, or Avi such as GH5 (EGs), GH3 (BGLs), and CE5 (AXEs) (Figure S5B and Table S1).

### 3.3. Predicted Cellulases, Hemicellulases, and Other Proteins

*T. erinaceum* displayed a diverse group of enzymes related to cellulose degradation with 16 different PCWDEs, including BGLs (5), EGs (5), CBHs (2), LPMOs (2), glucooligosaccharide oxidase (GOOX) (1), and cellobiose dehydrogenase (CDH) (1). Most of these PCWDEs were not identified on the secretome produced on Glc, except for one CBH (GH6) and two BGLs (GH3), in addition to one GOOX (AA7) detected on Glc and Avi.

As mentioned, cellulases CBHI (GH7) and CBHII (GH6) showed the highest secretion levels in SCS and ECB. CBHII was more secreted at 96 h (seq 603), while CBHI (seq 1908) showed higher spectrum counts at 120 h. Moreover, the CBHI spectrum counts were higher at 144 h in the secretome produced on ECB. A low concentration of EGs was detected, and the secretion profile was substrate-dependent. The total spectrum counts of EGs were higher at 96 h and 144 h in the secretome produced on SCS and lower at the last time point on ECB. Considering the BGLs, one secreted enzyme was identified in SCS and ECB with higher concentrations at 120 h. One LPMO was also produced at the time points tested but in a higher abundance at 96 h (ECB) and 120 h (SCS) (Table S1; Figure S6). Together, these data evidenced that the *T. erinaceum* secretome profile is dependent on the substrate and time of cultivation, and similar feedstocks with the same pretreatment induced the production of different PCWDEs.

Hemicellulose is a highly decorated polymer containing side chains that require enzymes to degrade the xylan backbone and accessory enzymes, e.g., mannanases, arabinofuranosidases, galactosidases, glucuronidases, and esterases. Twenty predicted hemicellulases were identified, especially in the *T. erinaceum* secretomes produced on SCS and ECB. In contrast, only four hemicellulases were identified on Glc, and six were detected in Avi (AXE, XLNs, xyloglucosyltransferase, and BXL). Four XLNs were identified in SCS and ECB, where seqs 7634 and 6279 represented the most abundant hemicellulases in the secretomes. These two XLNs belong to families GH10 (seq 7634) and GH11 (seq 6279) and showed different secretion profiles over time, with higher concentrations at 96 h. A greater diversity of CAZymes was evident in the secretomes produced on plant biomass when compared to Avi and Glc, with the identification of three AXEs (CE5), one AGL (GH27), three ABFs (GH43, GH54 and GH62), one β-L-arabinofuranosidase (GH127), one endo-α-1,5-L-arabinanase (GH43), one xyloglucanase (GH74), and one xyloglucosyltransferase (GH16).

Other enzymes that might not be directly related to biomass degradation were identified, such as a cutinase (CE5), which commonly acts in cutin, a polymer contained in waxes that compose plant cuticles in some species [83]. One endo-α-1,4-polygalactosaminidase (GH114) and ten mannosidases (α-1,6- mannanase, α-1,2- mannanase, β-1,3-mannanase, β-1,4-mannanase) were also found in the *T. erinaceum* secretomes. Considering the importance of some oxidases, we identified two peroxidases (AA2), one CDH (AA,3), a glyoxal oxidase (AA5), and a glucooligosaccharide oxidase (AA7). Enzymes with predicted function in fungal cell wall degradation (chitinases, exo-β-1,3-glucanases, exo-α-1,3-glucanase, β-1,3-glucanosyltransglycosylase), protein modification (transferases and peptidases), and fungal metabolism (esterases) without predicted signal peptides suggest an autolysis mechanism.

### 3.4. Enzymatic Profile of T. erinaceum Secretomes Produced on Plant Biomass

**Figure 6:**
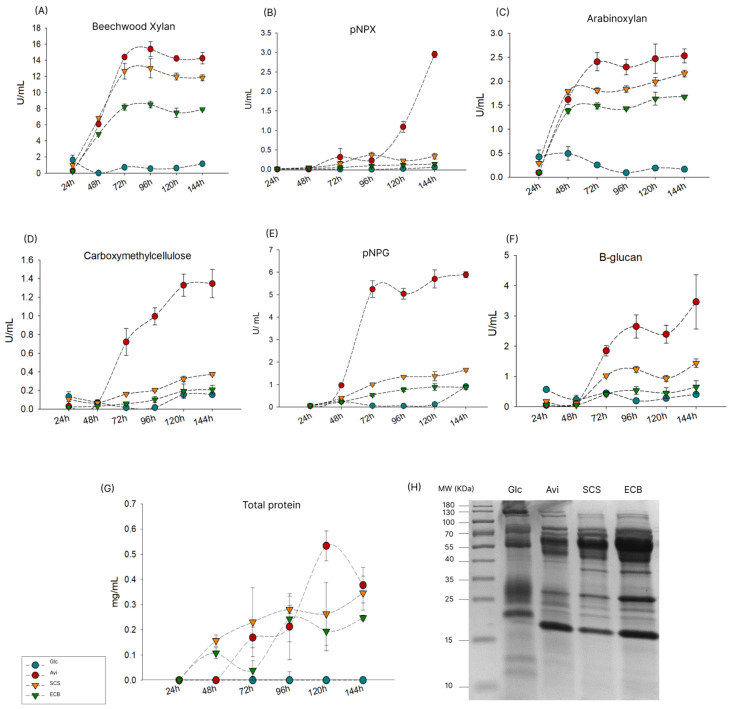
Enzymatic activities of *T. erinaceum* secretomes produced on glucose (Glc), avicel (Avi), pretreated sugarcane straw (SCS), and pretreated energy cane bagasse (ECB). The substrates used were (**A**) beechwood xylan, (**B**) carboxymethylcellulose, (**C**) *p*NPX, (**D**) *p*NPG, (**E**) arabinoxylan, (**F**) β-glucan, and (**G**) total proteins, measured with the Bradford method. One unit of enzyme activity was defined as the amount of enzyme releasing 1 µmol of product per minute. (**H**) SDS-PAGE 12% containing 10 µg of each secretome (144 h) to compare differences in protein profiles.

After studying the PCWDEs identified in the *T. erinaceum* secretomes by mass spectrometry, we also measured the activity of some enzyme groups over time (24–144 h) (Figure 6). The soluble substrates tested were beechwood xylan, CMC, arabinoxylan, β-glucan, *p*NPG, and *p*NPX. Overall, the secretome produced on Avi showed the highest activity, especially at 72 h, reaching 5.89 U/mL in *p*NPG, 1.34 U/mL in CMC, and 3.4 U/mL in β-glucan. The same activities were lower on SCS and ECB, despite their similar profiles. The secretomes produced on SCS and ECB showed higher activity on hemicellulose components (xylan and arabinoxylan), indicating these substrates are inducers of XLN secretion. XLN activity increased at 48 h in the secretomes produced on Avi, SCS and ECB, with the highest levels found from 72 h to 144 h. However, there was very low activity on *p*NPX, which increased in the secretomes produced on Avi at 144 h (2.95 U/mL). For further evaluation of the *T. erinaceum* secretomes, the total protein secreted was also measured. The highest secretion levels were observed at 120 h in the secretome produced on Avi (0.53 g/L).

### 3.5. β-Glucosidases Identified in the T. erinaceum Secretome

**Figure 7:**
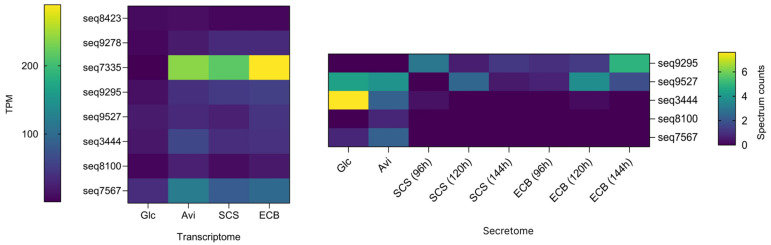
Quantification of β-glucosidases (BGLs) identified in *T. erinaceum* transcriptome and secretome. Samples were produced in different conditions: glucose (Glc), avicel (Avi), pretreated sugarcane straw (SCS), and pretreated energy cane bagasse (ECB). Enzyme quantification in the secretome was realized by normalized total spectrum counts and in the transcriptome by transcripts per million (TPM).

Considering that the *T. erinaceum* secretomes produced on plant biomass showed higher enzymatic activity in *p*NPG than *T. reesei* [45], we searched for BGLs in the *T. erinaceum* secretome and transcriptome data. Five BGLs classified in the GH3 family (seqs 9295, 9527, 3444, 7567, and 8100) were identified considering all secretomes. At a genomic level, these BGLs are duplicated, except for BGL seq8100, which is a single copy. The predicted BGL seq 9527 was identified in all the medium conditions, while the sequences 3444, 8100, and 7567 showed significant amounts only in the secretomes produced on Glc and Avi, and sequence 9295 was exclusively produced on SCS and ECB. The diversity of BGLs was higher in the transcriptome data, with eight GH3 sequences found in all carbon sources. Considering the secretomes, seq 9527 and seq 3444 were the BGLs most secreted by *T. erinaceum* (Figure 7). The predicted BGLs seq 3444 and seq 7567 showed higher similarity with *T. reesei* Cel3B (more than 85%), and seq 9295 showed 50% identity with Cel3E (Figure S7). Because *T. reesei* produces low levels of BGLs, and these enzymes are essential for producing glucose from cellobiose, also relieving CBHs cellobiose-dependent inhibition, the *T. erinaceum* BGLs found in the secretome were further analyzed and renamed as *Te*Bgl3A (seq 9295), *Te*Bgl3B (seq 3444), *Te*Bgl3C (seq 9527), *Te*Bgl3D (seq 7567), and *Te*Bgl3E (seq 8100). TeBgl3C showed the lowest identity with *T. reesei* sequence and then was selected for further recombinant production and characterization. *Te*Bgl3C has 97% identity with noncharacterized proteins from *T. gamsii* and *T. atroviride* but only 55% identity with the Cel3B from *T. reesei*. Additionally, TeBgl3C was the only beta-glucosidase identified by proteomics under all the conditions analyzed.

### 3.6. TeBgl3C Characterization

GH3 sequences from the previously characterized genera *Trichoderma*, *Penicillium*, *Aspergillus*, and *Talaromyces* were selected from the CAZy database and compared to the GH3 sequences found in the *T. erinaceum* secretome for phylogenetic analysis. The five *T. erinaceum* GH3 sequences found in the secretomes were clustered with BGLs, considering the GH3 family is represented by BGLs (EC 3.2.1.21) and BXLs (EC 3.2.1.37). *Te*Bgl3A and *Te*Bgl3E were grouped in the same clade as *Tr*Cel3A, while *Te*Bgl3B, *Te*Bgl3C, and *Te*Bgl3D shared phylogenetic similarities with *Tr*Cel3B (Figure S8).

**Figure 8:**
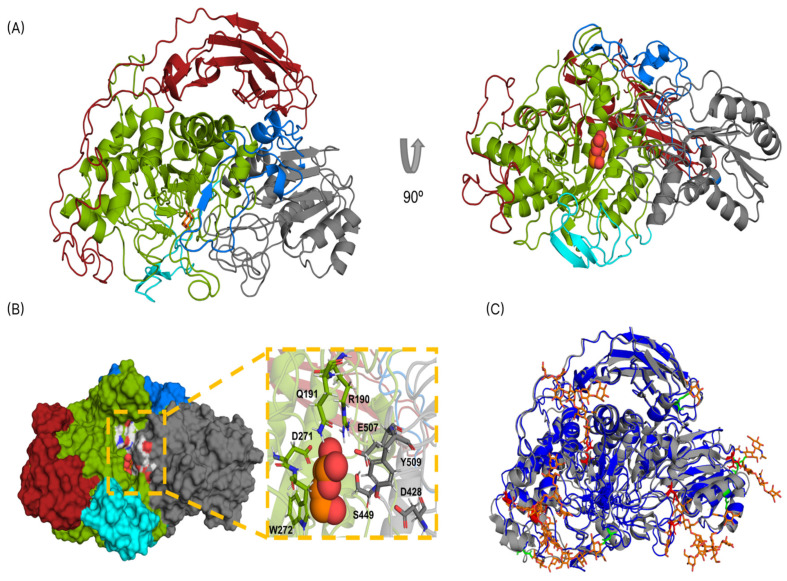
Structural modeling of *T. erinaceum Te*Bgl3C. (**A**) Schematic representation of *Te*Bgl3C showing three distinct folding domains: Domain I—TIM-barrel fold (green), Domain II—(α/β)_6_ sandwich (gray), and Domain III—FnIII-like (red), connected by two linker regions: Linker I—cyan and Linker II—marine blue. A glucose molecule was predicted to bind the active site of *Te*Bgl3C. (**B**) Surface representation showing the active site of *Te*Bgl3C. Residues in Domain I -1 subsite (green) and Domain II +1 and +2 subsites (gray). (**C**) Representation of *Te*Bgl3C overlapped with *Nc*Cel3A (id: 5NBS.A). Twelve N-glycan moieties were found in the crystallographic structure of 5NBS (orange) chain A. Five residues (green) represent N-glycosylation sites NXT/S only found in *Nc*Cel3A, and seven residues (red) are N-glyc sites shared by both structures.

*Te*Bgl3C (MW: 95 kDa; 865 aa) contains a nonspecific N-terminal (117–351 aa), a periplasmic bglx conserved domain with predicted function on carbohydrate transport and metabolism, a GH3 domain (412–651 aa; Pfam 00933), and a C-terminal fibronectin type III domain (787–855). *T. gamsii* GH3 (NCBI id: PON22580) shows the highest similarity (97%) with *Te*Bgl3C based on the NCBI database. However, no biochemical characterization has been reported so far. The *Te*Bgl3C structure was modeled with a high C score of 0.51 (−5 to 2), a predicted TM-score of 0.78 ± 0.1, and an estimated RMSD = 7.3 ± 4.2 Å. The *Rasamsonia emersonii* BGL (*Re*Cel3A) was the closest structure in the PDB database (4D0J.A). *Te*Bgl3C exhibits a higher estimated TM score (0.962) than *Re*Cel3A, an estimated RMSD = 0.84 Å, and a 96.8% sequence coverage, confirming the models’ high quality. *Te*Bgl3C also exhibited a moderate identity with other fungal GH3 enzymes in the PDB, such as the BGL *Nc*Cel3A from *Neurospora crassa* [84] (59%; id: 5NBS) and BGL1 from *Aspergillus aculeatus* [85] (56.7%; id: 4IIB). Therefore, the *Te*Bgl3C model presented all the hallmarks of a GH3 enzyme from the subcluster C2, which consisted of structures with three distinct folding domains (Figure 8A). Domain 1 is formed by the collapsed TIM-barrel fold [or ββ(β/α)_6_ fold] with several loops, which are connected by Linker 1 to the second domain. Domain 2 comprises the (α/β)_6_ sandwich with loops, which is well conserved among all the GH3 enzymes. Lastly, Domain 3 is a FnIII-like or immunoglobulin s-type domain, connected by Linker 2 to the second domain. This FnIII domain is a β-sandwich folding composed of two layers of β sheets, one with three and the other with four β strands, respectively. The C-terminal FnIII-like stabilizes the barrel and sandwich domains by the opposite side of the catalytic site. The function is unknown, but it has been suggested that a stabilization in TIM-barrel structure presents an incomplete fold by the FnIII domain [86]. Another important aspect involving FnIII is the suggestion that this domain interacts with lignin through electrostatic and hydrophobic interactions that can overcome the repulsive forces between the catalytic domain and lignin [87]. Furthermore, the substrate-binding site of *Te*Bgl3C was predicted with a moderate C score of 0.75 (0–1) and 65 clusters, based on homology using Cel3A from *T. reesei* as the template. The two *Te*Bgl3C catalytic residues predicted are D271, found in Domain 1 and reported as a nucleophile residue, and E507, the acid/base located in Domain 2. Some substrate-binding residues such as R190, Q191, and W272 were found in *Te*Bgl3C located at Domain 1, composing the -1 subsite, while residues D428, S449, and Y509 compose the +1 and +2 subsites and are situated in Domain 2 (Figure 8B). The crystal structure of the *N. crassa* BGL (*Nc*Cel3A) containing several N-linked glycans was superimposed with our model (Figure 8C). The analysis showed that *Te*Bgl3C has seven predicted N-glycosylation sites matching the twelve sites displayed by the *Nc*Cel3A structure, suggesting a distinct glycosylation pattern. The most frequent N-glycosylation sequon found for both models was Asn-X-Thr.

For recombinant production in *A. nidulans*, *Te*Bgl3C- and *Tr*Cel3B-encoding genes were cloned in an integrative cassette harboring a functional *pyrG* gene, two homologous arms for the locus of insertion (1 Kb each), a glyceraldehyde-3-phosphate dehydrogenase constitutive promoter (*Pgpda*), and a tryptophan biosynthesis gene terminator (*TtrpC*). The genes were integrated into the IS1 between the AN6638 and AN6639 genes found in chromosome I via homologous recombination [78]. The *Te*Bgl3C gene is 2595 bp long and encodes a 95 kDa protein with a theoretical isoelectric point (pI) of 6.0. The *Tr*Cel3B gene is 2622 bp long and encodes a 96 kDa enzyme with a theoretical pI of 6.15.

**Figure 9:**
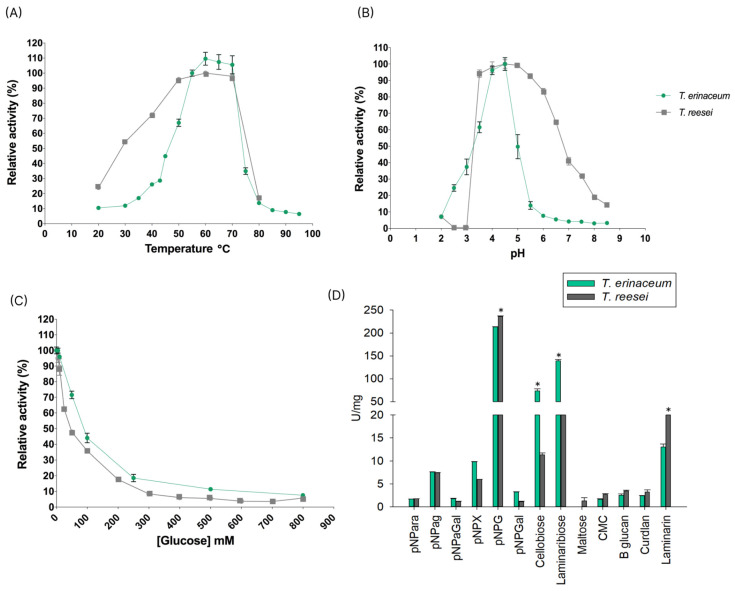
Biochemical parameters of recombinant *Te*Bgl3C and *Tr*Cel3B. (**A**) Optimum temperature (°C), (**B**) optimum pH, (**C**) glucose tolerance, and (**D**) substrate specificity. Assays (**A**–**C**) were performed using *p*NPG as substrate. Recombinant enzymes were incubated at 60℃ for 30 min, and enzymatic activities were converted to µmol of product per minute (U)/protein mg; *t*-test * *p*-value < 0.05.

The optimal temperature and pH of *Te*Bgl3C and *Tr*Cel3B were measured using *p*NPG (Figure 9A,B). Both enzymes exhibited maximal activity at 60 °C. However, they showed high enzymatic activity between 50 °C and 70 °C. At the optimal temperature, *Te*Bgl3C showed the highest activity at pH 4.5, but both enzymes presented a great performance between pH 4.0 and 5.0 (Figure 9B).

BGLs are frequently inhibited by glucose [88]. We performed assays using *p*NPG as the substrate, testing both enzymes in the presence of glucose. *Te*Bgl3C showed a slightly higher tolerance to glucose than *Tr*Cel3B in the range from 200 mM to 800 mM, with a reduction of 50% in enzymatic activity at 100 mM glucose (18 g/L) (Figure 9C). The substrate preferences of *Te*Bgl3C and *Tr*Cel3B were determined using 13 substrates, including 6 *p*-nitrophenyl derivatives (*p*NPG, *p*NPX, *p*NPαG, *p*NPαGal, *p*NPGal, *p*NPαAra), 3 disaccharides (cellobiose, laminaribiose, maltose), and 4 polysaccharides (CMC, β-glucan, curdlan, laminarin). *Te*Bgl3C and *Tr*Cel3B displayed the highest specific activities on *p*NPG with 213.26 and 235.94 U/mg, respectively. Low activities were found on *p*NPX and *p*NPαG for both enzymes. However, *Te*Bgl3C showed remarkable enzymatic activity on cellobiose (73.6 U/mg) and laminaribiose (139.43 U/mg), while *Tr*Cel3B had a higher activity on laminarin (23.5 U/mg) (Figure 9D).

Considering *Te*Bgl3C’s capacity to hydrolyze laminaribiose and cellobiose, we tested the enzyme activity using cellohexaose (β-1,4-C6) and laminarihexaose (β-1,3-L6) (Figure S9), analyzing the hydrolysis products by capillary electrophoresis. The enzyme showed a strong capacity to decompose oligosaccharides with six glucose molecules regardless of the linkage type (β-1,4 or β-1,3). Interestingly, the enzyme showed transglycosylation activity after 12 h of incubation with both substrates.

## 4. Discussion

The genetic engineering of fungal cell factories to produce high amounts of lignocellulolytic enzymes has taken years to achieve techno-economic viability. Filamentous fungi such as *T. reesei* have a superior capacity to produce cellulases, and some mutant strains have been used for enzyme production at an industrial scale [89,90]. The biological processes contributing to enzyme secretion yields, such as nutrient sensing, transcriptional regulation, translation, and secretory pathway, must be considered to develop a microbial cell factory for PCWDE production [91].

The nutritional requirements and enzyme regulation demonstrate that fungi might employ different approaches for hydrolyzing plant biomass. *T. reesei* secretome, for example, is focused on attacking the central structure of cellulose microfibrils [92], while *T. harzianum* is considered more efficient for proteases and chitinase production [93]. *T. erinaceum* has a versatile genome, including many interesting PCWDEs for lignocellulose degradation, and its enzymatic capacity was demonstrated in a broad range of soluble substrates. Additionally, the transcriptome and secretome under different conditions showed that plant biomass induces CBH secretion (seq 603 and 1908), representing approximately 70% of the total cellulases. In this context, it is important to consider that seq 1908 displays 77% identity with *T. reesei* orthologous Cel7A, while Cel6A is 78% identical to seq 603. These enzymes represent 80% of the cellulases produced by *T. reesei* [94], a pattern very similar to that of *T. erinaceum*, even though these fungi do not belong to the same phylogenetic clade according to our analysis. Nevertheless, *T. erinaceum* also displayed an expanded hemicellulase repertoire. The fungus produced a great diversity of hemicellulases on SCS and ECB, and two XLNs (seq 6279 and seq 7634) represented around 35% of the total CAZymes, similar to CBHs (40% of the secreted CAZymes).

Previous works that explored *Trichoderma* spp. and *Aspergillus* spp. capacity for hemicellulose degradation, have suggested more efficient lignocellulosic hydrolysis using a mixture of *A. niger* and *T. reesei* secretomes due to the specialization of *A. niger* or *A. nidulans* in producing XLNs [92,95,96]. At the same time, *T. reesei* is known for cellulase production [97]. Apart from this, the *T. erinaceum* secretome represents a nonspecific enzymatic cocktail enriched with both groups of hemicellulases and cellulases, whose levels were adjusted according to the time course of cultivation.

Transcription factors are central hubs of gene regulation. Many TFs are conserved among the filamentous *Ascomycota* group, such as *clr1/2*, *mcmA*, *bglR*, *ace1*, *cre1*, and *xyr1*. These TFs were described in model systems, e.g., *Aspergillus* spp., *N. crassa*, and *T. reesei*. Moreover, some are specifically involved in cellulose degradation, indicating overlapping functions or a well-coordinated regulatory process with fine tuning depending on the species [42]. Several plant biomass components can trigger transcriptional activation or the repression of PCWDEs. Previous studies showed that glucose activates the TF CRE1, which represses the expression of PCWDEs genes through carbon catabolite repression (CCR) [98]. This regulatory system prevents energy waste by producing extracellular enzymes only when needed by the metabolic pathways. The regulation of *T. reesei* cellulase gene expression involves additional transcription factors, including the activators ACE2 and ACE3. In the presence of lignocellulose and absence of glucose, the expression of PCWDEs genes is activated by XYR1 [40,99], ACE2, and ACE3 [39,100], allowing polysaccharides’ digestion and release of simple sugars, representing a highly accurate regulation process [101,102,103]. The regulator XYR1 is involved in D-xylose catabolism and xylan degradation in most fungi. In *Aspergilli* and *T. reesei*, it also controls the cellulolytic system. In *T. erinaceum*, XYR1 seems to play a similar role based on its expression pattern and sequence similarity to the *T. reesei* TF. The activator ACE2, when deleted in *T. reesei*, reduces the expression of the main cellulases but is overexpressed in the presence of sophorose [73]. In *T. erinaceum*, the *ace2-*predicted homolog’s expression was lower than the other TFs analyzed. ACE3, as well as ACE2, is a positive regulator of some cellulases and xylanases. In *T. erinaceum*, it shows clear regulation through its overexpression in the presence of Avi, SCS, and ECB.

In contrast, ACE1 acts as a repressor for cellulase and xylanase production. Its deletion in *T. reesei* resulted in increased cellulases and hemicellulases production. Homologs of this TF were described in *Ascomycota*, and its deletion in *T. reesei* increased the expression of main cellulases (*cbh1*, *cbh2*, *egl1*, and *egl2*) and hemicellulases. ACE1 is induced by lactose and repressed by CRE1. In *T. erinaceum*, the predicted ACE1 homolog was not induced by any carbon source, and the CRE1 homolog is overexpressed mainly in SCS. CRE1 plays a fundamental role in fungal development, sugar uptake, and hyphal development. Its deletion can negatively interfere with cellulase secretion [42,98].

Orthologues of these master regulators were identified in the *T. erinaceum* dataset, in addition to seven predicted and nonannotated TFs with a conserved domain present in important fungal TFs such as XLNR and GAL4 (pfam 04082). The characterization of TFs is necessary to understand the regulatory network of PCWDEs in filamentous fungi, which seems far more complex [103,104,105,106].

*T. erinaceum* can be explored as a fungal cell factory for PCWDE production and as a source of specific enzymes deficient in other cocktails, such as the low BGL activity in *T. reesei* [52,107,108]. Enzymatic cocktail supplementation with BGL was previously described, and the supplementation of Novozyme-188 with Spezyme-CP improved the glucan digestibility of pretreated switchgrass [109]. Moreover, the *T. reesei* RUT C30 secretome supplemented with BGL from *A. niger* increased the hydrolysis yield of steam-exploded corn stover by 80.93% [110], and an increase of 40% of sugar cane bagasse hydrolysis was achieved by supplementing a *T. reesei* cocktail with EG from *Bacillus subtilis* and BGL from *A. niger* [111]. This effect has been extensively studied in enzymatic cocktails, and BGLs play a positive role in hydrolysis, reducing cellobiose and cellooligomer concentration, alleviating the inhibition of CBHs and EGs by their products [112].

Considering the BGLs found in the *T. erinaceum* secretomes, *Te*Bgl3C was selected for further studies because this enzyme was secreted in all the conditions tested (Glc, Avi, ECB, and SCS) and showed 55% identity with high coverage (100%) compared to *T. reesei Tr*Cel3B. *Te*BglC displayed higher activities on *p*NPG, cellobiose, and laminaribiose compared with *Tr*Cel3B and oligomers containing β-1,4 or β-1,3-linked glucose.

Preliminary data also suggest that *Te*Bgl3C displays transglycosylation activity using C6 and L6 as substrates. This activity is important considering the cellulase induction system described in *T. reesei*. Some BGLs have been identified as responsible for catalyzing transglycosylation reactions, converting cellobiose to cellotriose and isomeric glucobiose. Cellobiose is essential for cellulase induction, and positional isomers such as 1,2-β-glucobiose (sophorose), 1,6-β-glucobiose (gentiobiose), and 1,3-β-glucobiose (laminaribiose) have shown higher induction capacity, evidencing the importance of transglycosylation for the regulation of cellulase expression [113].

## 5. Conclusions

The aim of this study was to investigate the metabolic capabilities and range of plant cell wall-degrading enzymes (PCWDEs) secreted by *T. erinaceum*. The secretome of *T. erinaceum* exhibited a similar proportion of typical cellulases and hemicellulases, such as CBHs and XLNs. The strain showed versatility in metabolizing various substrates, potentially due to the presence of multiple sugar transporters and a conserved mechanism for the transcriptional regulation of PCWDEs, including some understudied transcription factors. The master regulators previously described in *T. reesei* were also identified in *T. erinaceum*, showing a similar regulation pattern depending on the inducers and carbon sources, except for ACE2. Furthermore, *Te*BglC from *T. erinaceum* demonstrated higher efficiency in hydrolyzing cellobiose (β-1,4) and laminaribiose (β-1,3), suggesting this fungus could be a valuable genetic resource for enzyme cocktail development. Given its biotechnological potential, further investigation using genetic engineering tools could enhance the secretion of PCWDEs by *T. erinaceum*.

## Data Availability

The *T. erinaceum* (named *Trichoderma erinaceum* F3) genome and transcriptome are available from NCBI under the BioProject ID PRJNA1073939. The secretome raw data, with the identifier PXD051248, are available from ProteomeXchange.

## Supplementary Materials

The following supporting information can be downloaded at: https://www.mdpi.com/article/10.3390/jof10060407/s1, Figure S1: BUSCO results for the assembly of the *Trichoderma erinaceum* genome; Figure S2: Secretome and transcriptome data correlation analysis; Figure S3: Principal component analysis (PCA) of gene expression profiles from *Trichoderma erinaceum*; Figure S4: Gene network coexpression analysis of *T. erinaceum* transcriptomes; Figure S5: Quantification of normalized spectrum counts and predicted CAZymes in *T. erinaceum*; Figure S6: The major cellulases produced in the *T. erinaceum* secretomes; Figure S7: Heatmap representing the GH3 enzymes identified in *T. erinaceum* transcriptome compared with the GH3 sequences from *T. reesei*; Figure S8: Phylogenetic analysis of characterized GH3 sequences retrieved from the CAZy database and predicted sequences found in *T. erinaceum* secretomes; Figure S9: Oligomers released after the digestion of cellohexaose (β-1,4-C6) and laminarihexaose (β-1,3-L6) by *Te*Bgl3C; Table S1: Number of important genes (genome data), full secretome of *T. erinaceum*, and lignocellulosic biomass characterization.
