# Supplementary material for: A Multiomics Perspective on Plant Cell Wall-Degrading Enzyme Production: Insights from the Unexploited Fungus Trichoderma erinaceum
Source: J Fungi (Basel). 2024 Jun 5;10(6):407. doi: 10.3390/jof10060407 (PMC11205114; doi:10.3390/jof10060407)
Supplement: Supplementary file 1 [file jof-10-00407-s001.zip › Supplementary_Material_JoF.pdf]

A multiomics perspective on plant cell wall degrading- enzymes production: insights from the unexploited fungus *Trichoderma erinaceum*

Michelle A. de Assis [1], Jovanderson J. B. da Silva [2], Lucas M. de Carvalho [2], Lucas S. Parreiras [2], João Paulo L. Franco Cairo [1,3], Marina P. Marone [2], Thiago A. Gonçalves [1], Desireé S. Silva [4], Miriam Dantzger [2], Fernanda L. de Figueiredo [1], Marcelo F. Carazzolle [2], Gonçalo A. G. Pereira [2] and André Damasio [1*]

1 Laboratory of Enzymology and Molecular Biology (LEBIMO), Department of Biochemistry and Tissue Biology, Universidade Estadual de Campinas (UNICAMP), Campinas, São Paulo, 13083-862, Brazil; mialexandrino@gmail.com (M.A.d.A.); joao.lourencofrancocairo@york.ac.uk jpcairo@gmail.com (J.P.L.F.C.); tikogoncalvesta@gmail.com (T.A.G.) ; fernandalfigueiredo@gmail.com (F.L.d.F.)

2 Genomics and BioEnergy Laboratory (LGE), Department of Genetics, Evolution, Microbiology and Immunology, Universidade Estadual de Campinas (UNICAMP), Campinas, São Paulo, 13083-862, Brazil; jovanderson14@hotmail.com (J.J.B.d.S.); lucasmigueel@gmail.com (L.M.d.C.); lucparreiras@gmail.com (L.S.P.); marina.marone@gmail.com (M.P.M.); mdantzger@gmail.com (M.D.); marcelo.carazzolle@gmail.com (M.F.C.); goncalo@unicamp.br (G.A.G.P.)

3 York Structural Biology Laboratory (YSBL), Department of Chemistry, University of York, York, YO10 5DD, United Kigdom; joao.lourencofrancocairo@york.ac.uk (J.P.L.F.C.)

4 SENAI Institute for Biomass Innovation, Três Lagoas, Mato Grosso do Sul, 79640-250, Brazil; d.silva@ms.senai.br

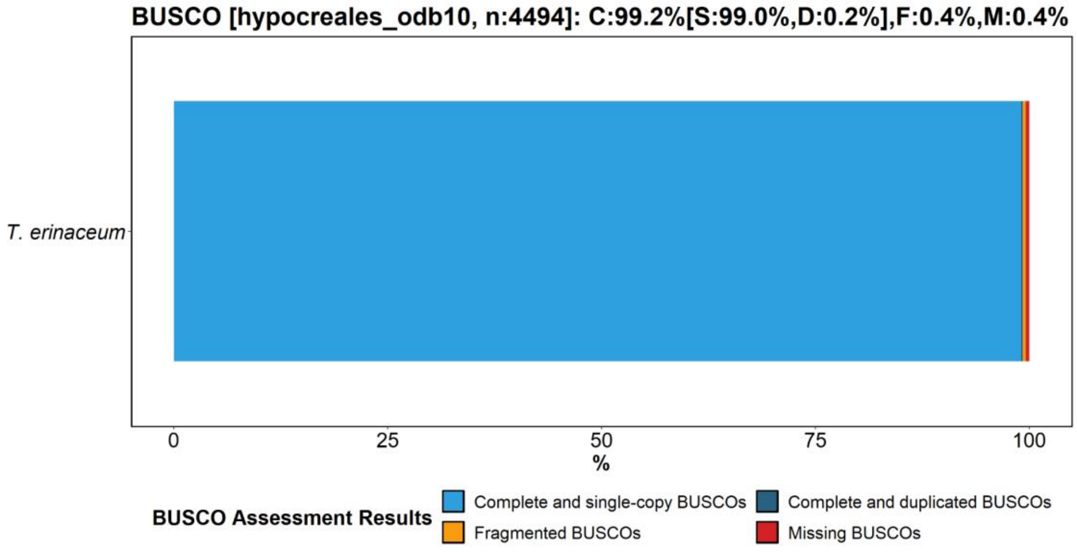

Fig. S1. BUSCO results for the assembly of the *Trichoderma erinaceum* genome. Complete (C) and single-copy (S) genes are shown in light blue, complete and duplicated (D) genes are shown in dark blue, fragmented (F) genes are shown in yellow, and missing (M) genes are shown in red. The BUSCO dataset of the hypocreales_odb10 including 4494 BUSCOs was used to assess assembly.

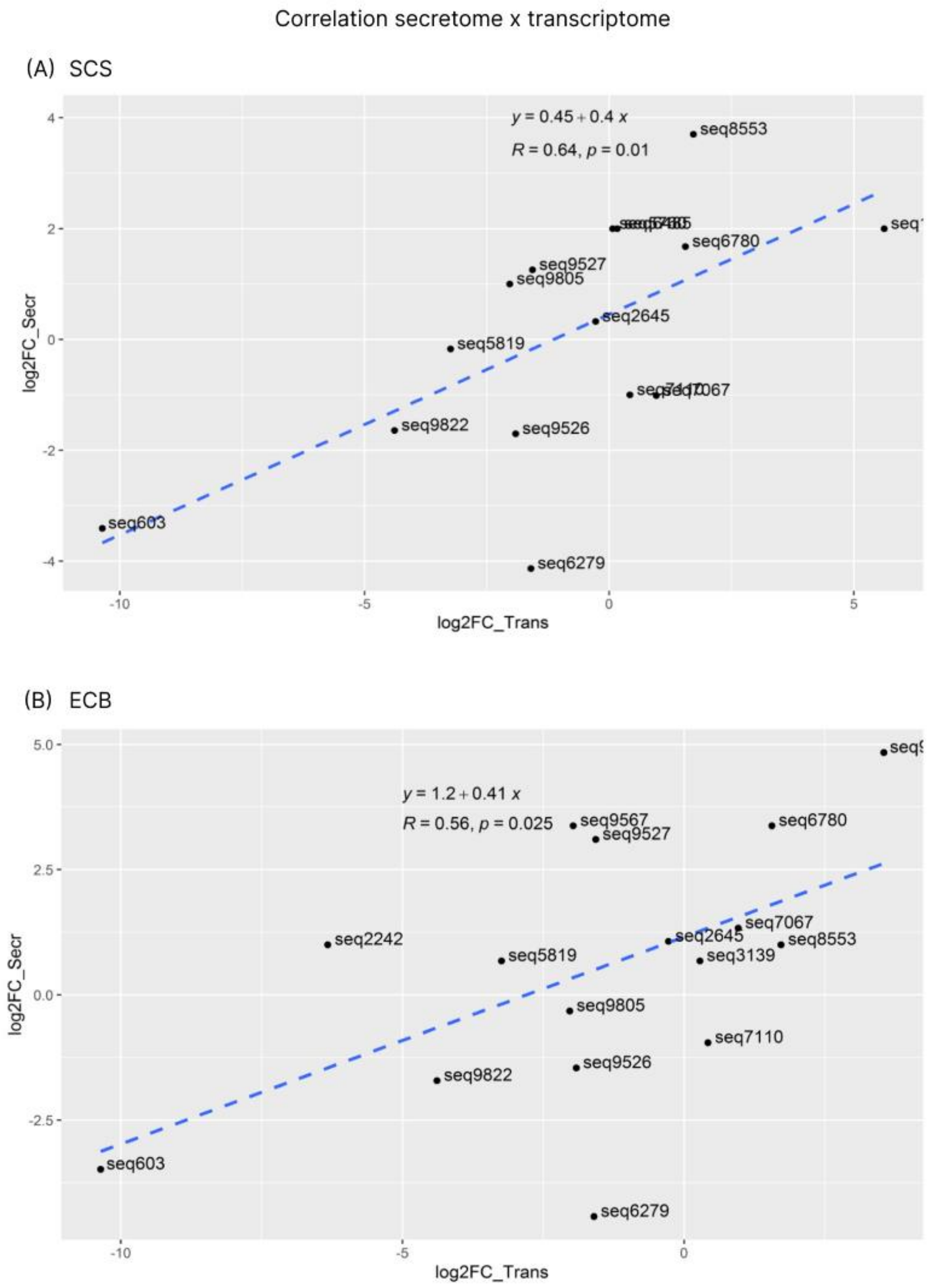

Fig. S2. Secretome (y axis) and transcriptome data (x-axis) correlation analysis based on log2 fold-change (FC). The FC was based on initial values from glucose analysis and (A) sugarcane straw (SCS) or (B) energy cane bagasse as the final values. The ratios were performed using transcripts per kilobase million (TPM) for transcriptome data and normalized spectrum counts for the secretome.

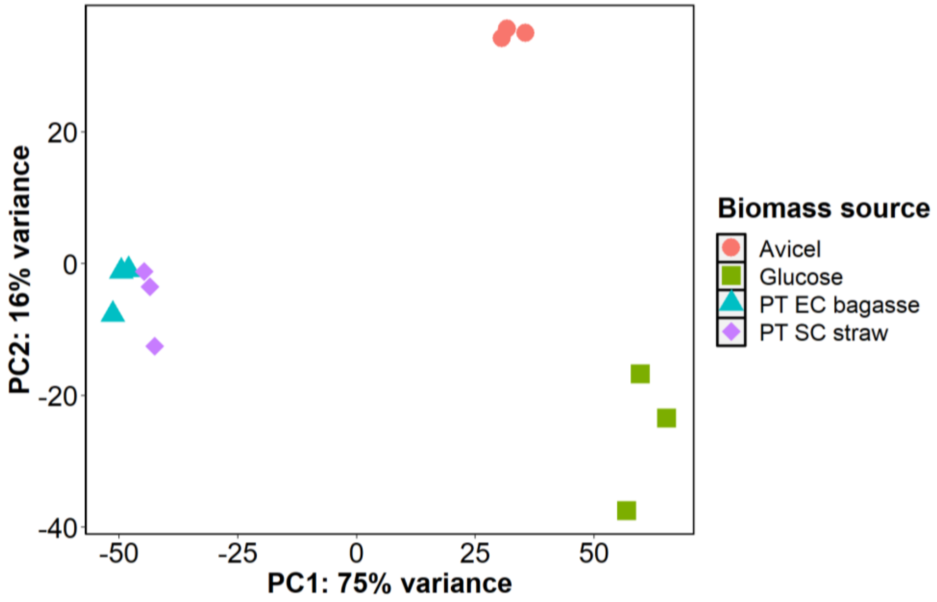

Fig. S3. Principal Component Analysis (PCA) of gene expression profiles from *Trichoderma erinaceum* grown under different biomass sources, including Avicel, glucose, pretreated energy-cane bagasse (PT EC bagasse), and pre-treated sugarcane straw (PT SC straw).

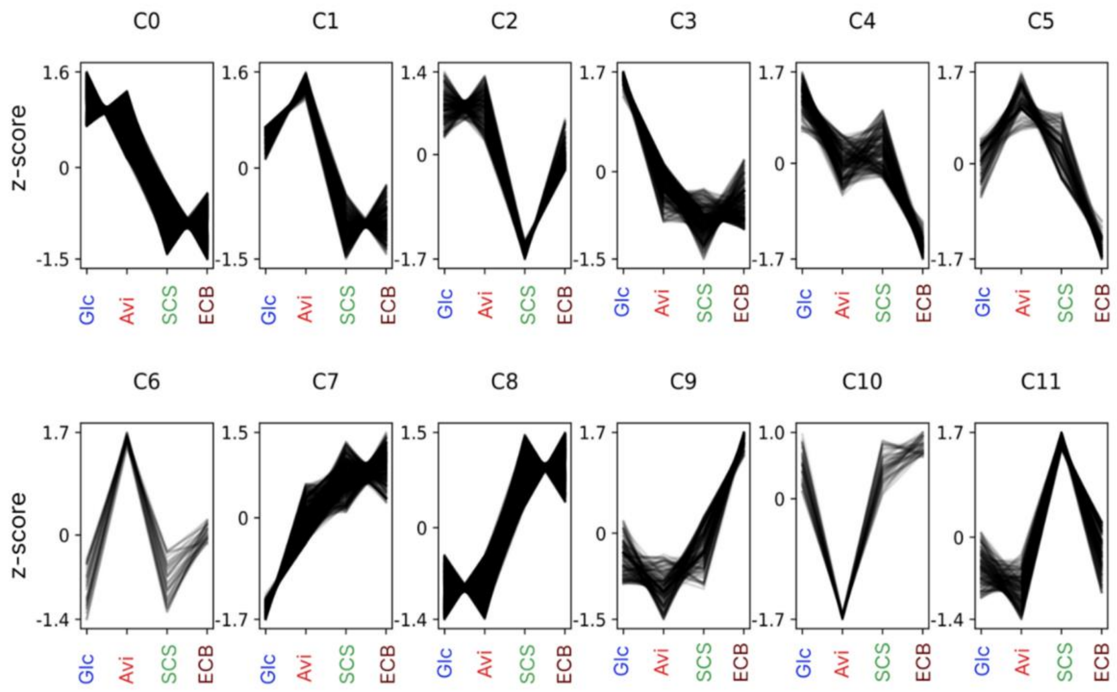

Fig. S4. Gene network co-expression analysis of *T. erinaceum* transcriptome data (Glc- glucose, Avi- avicel, SCS-pretreated sugarcane straw, and ECB– pretreated energy cane bagasse). Genes were grouped into 12 clusters. The GO enrichment analysis of differentially expressed genes (DEG) was performed using the ShinyGO web platform with a cut-off of *FDR <= 0.05* for significant processes.

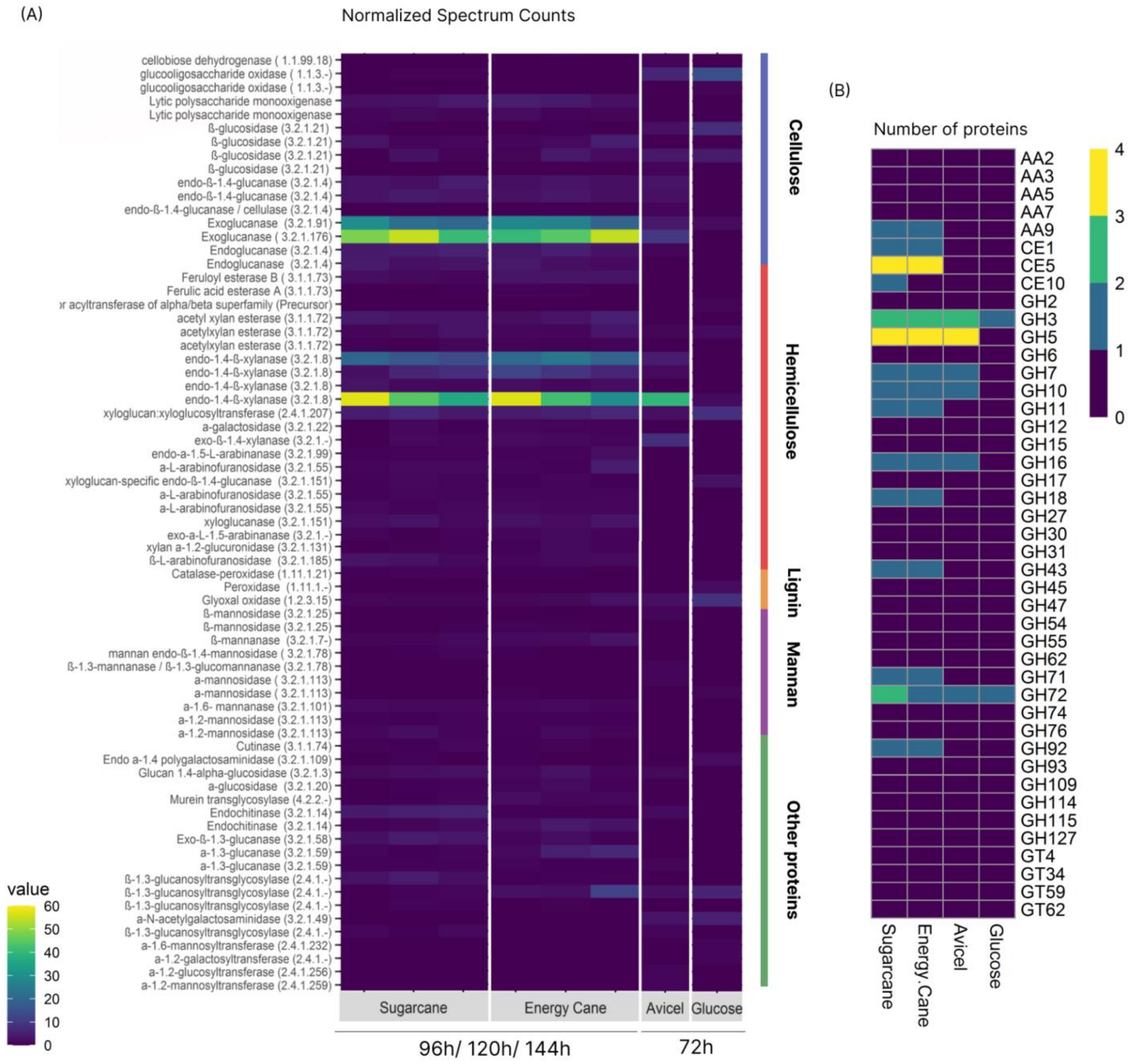

Fig. S5. Quantification of normalized spectrum counts and predicted CAZymes. (A) Normalized spectrum count of predicted enzymes identified in the secretomes produced on sugarcane, energy cane, Avicel, and glucose. The spectrum counts normalization considers three parameters: the total number of spectra in each biosample (t), the average number of spectra across all biosamples (μ), and the multiplication of each spectrum count (s) in each biosample by the average count over the total spectrum count [s*(μ/t)]. (B) Predicted enzymes grouped according to their CAZy families.

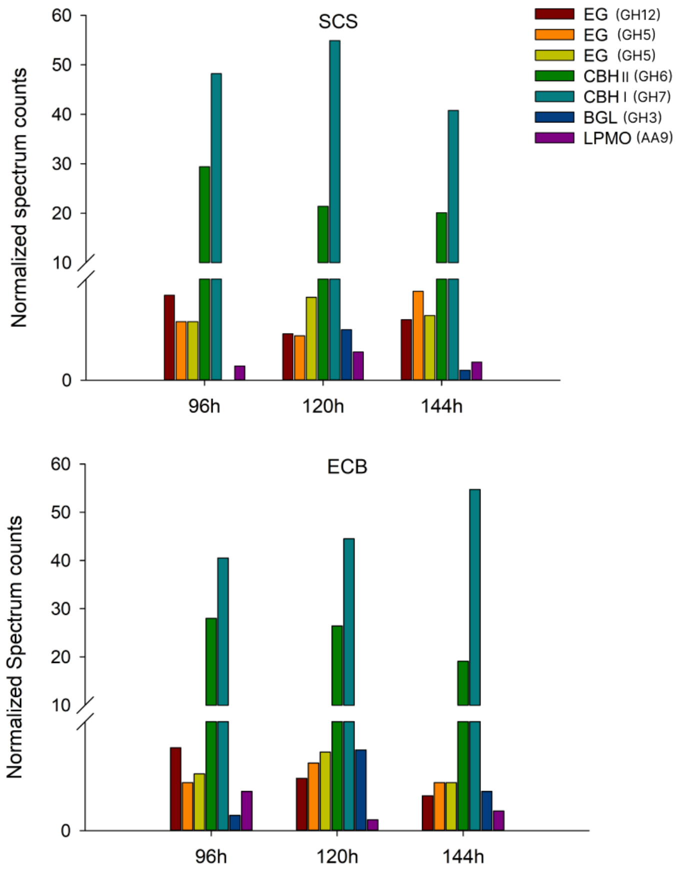

Fig. S6. The major cellulases in the *T. erinaceum* secretomes were produced on plant biomass. Normalized spectrum counts of three endoglucanases (EGLs), two cellobiohydrolases (CBHs), one β-glucosidase (BGL), and one lytic polysaccharide monooxygenases (LPMO) identified in the secretomes of *T. erinaceum* produced on pretreated sugarcane straw (SCS) and pretreated energy cane bagasse (ECB).

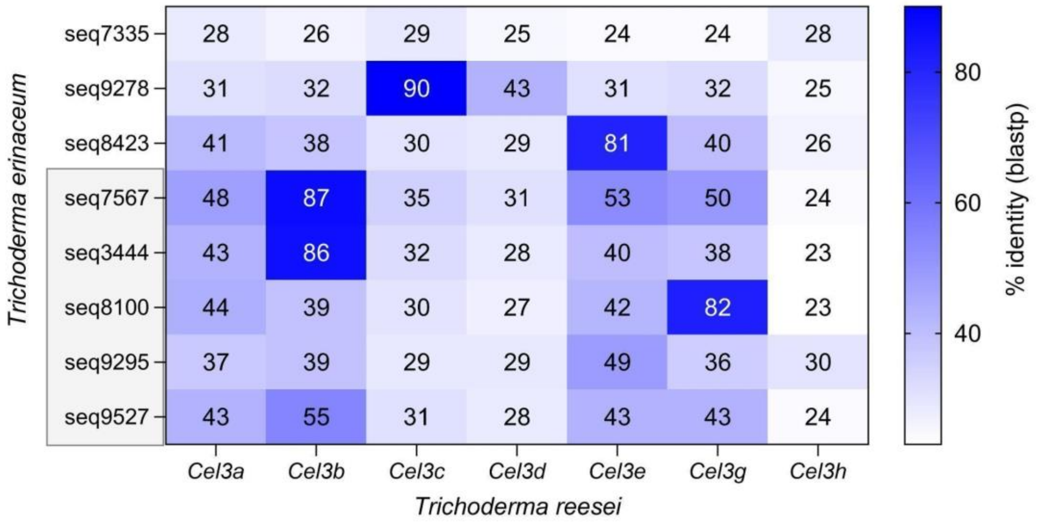

Fig. S7. Heatmap representing all the GH3 predicted enzymes identified in *T. erinaceum* transcriptome (left) compared with the GH3 sequences from *T. reesei* (Cel3A-H). The alignments were performed using Blastp (protein-protein Blast). Sequences identified in the secretome are indicated in the gray box.

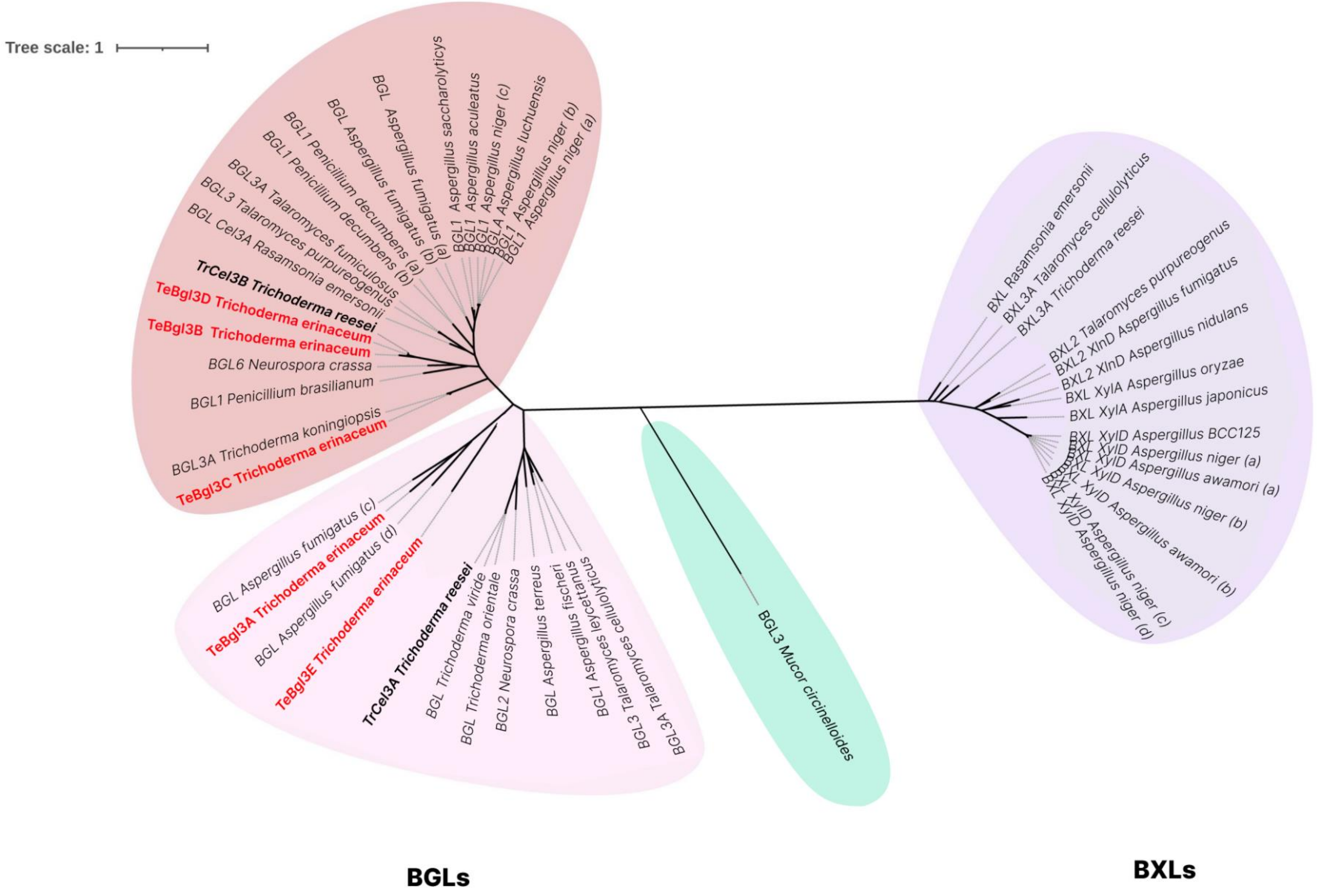

Fig. S8. Phylogenetic analysis of characterized GH3 sequences retrieved from the CAZy database and predicted sequences found in *T. erinaceum* secretomes. *Te*Bgl3A (seq9295) was grouped in the same clade of *Tr*Cel3A, and *Te*Bgl3E (seq8100) (pink), while the *Te*Bgl3B (seq3444), *Te*Bgl3D (seq7567), and *Te*Bgl3C (seq9527) grouped with *Tr*Cel3B (red). The multiple alignment was performed using T-COFFEE (Expresso protein alignment) server with default parameters and without curation. Maximum likelihood analysis was performed to classify the GH3 CDSs sequences as BGLs (red and pink clades), BXLs (gray clade), and BGL glucose tolerance (blue clade). The phylogenetic tree was drawn using the iTOL web platform.

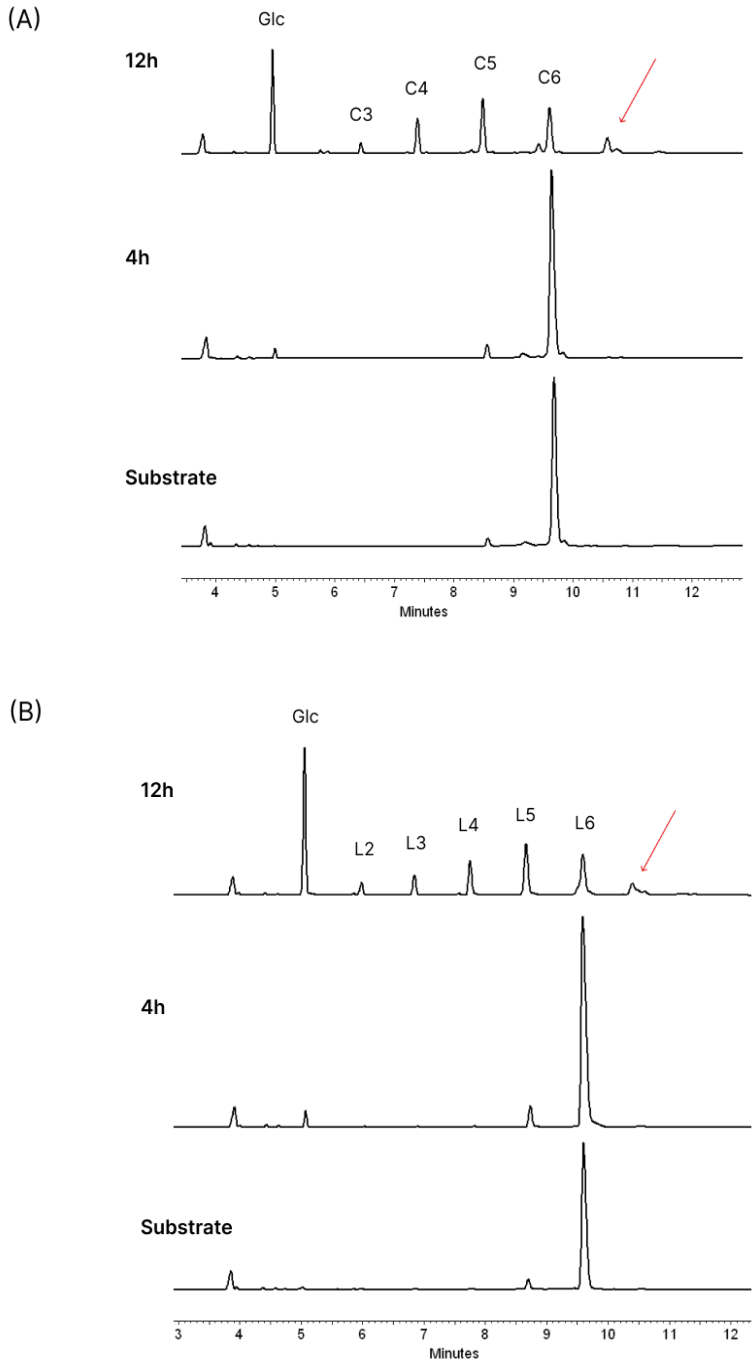

Fig. S9. Oligomers released after the digestion of (A) cellohexaose (β-1,4-C6) and (B) laminarihexaose (β-1,3-L6) by *Te*Bgl3C. Red arrows indicate peaks that might suggest transglycosylation activity. The reactions were incubated for 4 or 12 hours at 40 °C with 6 ng/µL of enzyme and 0.2 mg/mL of oligosaccharides (L6 and C6, 4.5 mM). Products were derivatized by reductive amination with 8-aminopyrene-1,3,6-trisulfonic (APTS). Separation was performed in a neutral capillary (P/ACE MDQ system; Beckman Coulter) with 50 µm in internal diameter and 50 cm in length at 20 kV/70–100 µA in 40 mM potassium phosphate buffer (pH 2.5), with the cathode in the inlet. The molecules labeled with APTS were excited at 488 nm, and emissions were collected in a 520-nm bandpass filter.
